# Evaluation of 1-Year vs Shorter Durations of Adjuvant Trastuzumab Among Patients With Early Breast Cancer: An Individual Participant Data and Trial-Level Meta-analysis

**DOI:** 10.1001/jamanetworkopen.2020.11777

**Published:** 2020-08-24

**Authors:** Seema Gulia, Sadhana Kannan, Rajendra Badwe, Sudeep Gupta

**Affiliations:** 1 Department of Medical Oncology, Tata Memorial Centre, Homi Bhabha National Institute, Mumbai, India; 2 Department of Biostatistics, Tata Memorial Centre, Homi Bhabha National Institute, Mumbai, India; 3 Department of Surgical Oncology, Tata Memorial Centre, Homi Bhabha National Institute, Mumbai, India

## Abstract

**Importance:**

The optimum duration of adjuvant trastuzumab among patients with early breast cancer is uncertain but of great therapeutic relevance.

**Objective:**

To compare shorter durations with 1 year of adjuvant trastuzumab for patients with early breast cancer.

**Data Sources:**

PubMed, Embase, Cochrane Central Register of Clinical Trials, and conference proceedings were searched from January 1, 2005, to June 30, 2019, for relevant randomized clinical trials (RCTs).

**Study Selection:**

To be eligible, the trial had to be randomized, compare a shorter duration with 1 year of trastuzumab as adjuvant treatment, and include patients with early breast cancer.

**Data Extraction and Synthesis:**

Individual patient data for disease-free survival (DFS) and overall survival (OS) were extracted from published survival curves of included RCTs; DFS and OS curves for each trial and the combined population were reconstructed. The DFS and OS hazard ratios (HRs) were estimated from the reconstructed survival curves as well as published estimates. The HR for DFS was used to test noninferiority using the median noninferiority margin of eligible RCTs. This study followed Preferred Reporting Items for Systematic Reviews and Meta-analyses reporting guideline.

**Main Outcomes and Measures:**

The primary outcome was DFS; OS and cardiac toxic effects were secondary outcomes.

**Results:**

There were 6 eligible RCTs with a median DFS noninferiority margin of 1.3 (range, 1.15-1.53), 5 of which had extractable individual patient data for 11 376 patients, 1659 DFS events, and 871 deaths. For shorter duration vs 1 year of trastuzumab, the 5-year DFS was 85.42% (95% CI, 84.41%-86.38%) vs 87.12% (95% CI, 86.15%-88.02%) (HR, 1.14; 95% CI, 1.03-1.25, 1-sided *P* for noninferiority = .004), and OS was 92.39% (95% CI, 91.61%-93.10%) vs 93.46% (95% CI, 92.73%-94.13%) (HR, 1.17; 95% CI, 1.02-1.33). Using trial-level published estimates from 6 RCTs, including 11 603 patients, 1760 DFS events, and 930 deaths, the HR for DFS was 1.15 (95% CI, 1.04-1.26; 1-sided *P* for noninferiority = .002) and, for OS, 1.17 (95% CI, 1.03-1.33). There was significantly less risk of congestive heart failure with shorter-duration trastuzumab (relative risk, 0.53; 95% CI, 0.38-0.74).

**Conclusions and Relevance:**

In this study, a shorter duration of adjuvant trastuzumab was noninferior to its 1-year administration and resulted in lower rates of cardiac toxic effects. These results suggest that a shorter duration may be the preferred option for patients with low-risk disease or a predisposition to cardiac toxic effects.

## Key Points

**Question:**

Is shorter duration adjuvant trastuzumab noninferior to its 1-year use for patients with early breast cancer?

**Findings:**

In this meta-analysis of individual patient data from 5 randomized clinical trials (with 11 376 participants) and trial-level data from 6 randomized clinical trials (with 11 603 participants), shorter duration of adjuvant trastuzumab was noninferior to 1 year of treatment in terms of disease-free survival and was associated with lower rates of cardiac toxic effects.

**Meaning:**

The findings of this study suggest that a shorter duration of adjuvant trastuzumab may be the preferred option for patients with low-risk disease or a predisposition to cardiac toxic effects.

## Teaser

This meta-analysis compares shorter duration with 1 year of adjuvant trastuzumab for patients with early breast cancer.

## Introduction

The standard adjuvant treatment *ERBB2* (formerly *HER2* or *HER2*/neu)-positive breast cancer includes chemotherapy and 1 year of trastuzumab, a recombinant, humanized monoclonal antibody that targets the ERBB2 receptor. Pivotal trials^1,2,3^ and subsequent collated evidence^4^ showed statistically significant and clinically meaningful reductions in risk of recurrence and death with the use of adjuvant trastuzumab. All randomized clinical trials (RCTs) except one^5^ included 1 year of trastuzumab in their experimental group, but notably, one^6^ failed to prove the superiority of 2 years of trastuzumab compared with 1 year. Also of note, the magnitude of benefit in the small FinHer trial,^5^ in which trastuzumab was administered for 9 weeks, appeared similar to that observed in trials using 1 year of administration. The choice of 1-year duration of adjuvant trastuzumab was somewhat arbitrary without strong preclinical or clinical rationale.^7^

Although trastuzumab is a well-tolerated drug, it is associated with cardiac dysfunction.^8^ An overview analysis found increased risk of congestive heart failure (CHF) (relative risk [RR], 5.1) with trastuzumab-based regimens (2.5%) compared with nontrastuzumab regimens (0.4%),^4,9^ and a meta-analysis^10^ showed that the absolute risk of high-grade CHF with 1 year of adjuvant trastuzumab was 1.4% vs no detectable risk with a 9-week course of trastuzumab (RR, 3.2).^10^

Based on these considerations, several RCTs have compared 1-year adjuvant trastuzumab with shorter treatment durations. Overall, 3 trials (SOLD, Short-HER, and E 2198)^11,12,13^ have compared ultrashort durations (9-12 weeks) of trastuzumab given concomitantly with chemotherapy while 3 other trials (PHARE, HORG, and PERSEPHONE)^14,15,16,17^ compared 6 months trastuzumab with 12 months. Individually, none of these trials, except PERSEPHONE,^17^ have proven noninferiority of shorter duration of trastuzumab according to their respective predefined noninferiority limits.

Because adjuvant trastuzumab duration is an important ongoing question with sufficient randomized evidence, we conducted a meta-analysis to investigate this question. Some previous meta-analyses evaluating the duration question have been reported.^18,19,20,21^ These analyses used trial-level data and did not include the updated PHARE^16^ and PERSEPHONE^17^ data. We have used a unique, recently reported method that is able to extract individual patient time-to-event data from published Kaplan-Meier survival curves^22^ and have synthesized the extracted data of RCTs to produce an individual patient data (IPD) meta-analysis for the trastuzumab duration question. We have included the updated PHARE^16^ and recently published PERSEPHONE^17^ data in this analysis. We report here the IPD meta-analysis and also a trial-level meta-analysis of all reported RCTs that have compared 1 year of adjuvant trastuzumab vs shorter durations.

## Methods

We performed IPD and trial-level meta-analyses for the trastuzumab duration question by extracting and synthesizing data from relevant RCTs. We followed the Preferred Reporting Items for Systematic Reviews and Meta-analyses (PRISMA) reporting guideline for this meta-analysis.

### Types of Studies

To be eligible, the trial had to be randomized, compare short duration (<1 year) with 1 year of trastuzumab as adjuvant treatment, and include patients with early (nonmetastatic) breast cancer. We excluded single-group prospective studies, retrospective analyses, and trials that included patients with advanced stage disease.

### Search Strategy

We identified eligible trials using a computerized search of the following databases from January 1, 2005, to June 30, 2019: PubMed, Embase, and the Cochrane Library. We also searched abstracts and virtual meeting presentations of the following major oncology conferences: American Society of Clinical Oncology Annual Meetings, 2005 to 2018; European Society of Medical Oncology/European Cancer Organization Meetings, 2005 to 2018; and San Antonio Breast Cancer Symposium Annual Meetings, 2005 to 2018. We also reviewed the references of the articles finally included in the analysis. The search strategy was as follows: *breast neoplasms* (Medical Subject Headings) AND *adjuvant* (text words) AND *trastuzumab* (Medical Subject Headings) OR *herceptin* (title and abstract) AND *duration* (title and abstract) AND *randomised or randomized controlled trials*. Two investigators (S. Gulia and S.K.) independently reviewed the titles, abstracts, and full texts to choose potentially relevant studies. Any disagreements were resolved with discussion among them and the corresponding author (S. Gupta).

### Data Extraction

Two of us (S. Gulia and S.K.) independently extracted the data, and disagreements, if any, were resolved by discussion among them and the corresponding author (S. Gupta). The following information was extracted from each selected trial: authors, publication year, number of patients in the experimental (ie, <1 year trastuzumab) and control (ie, 1 year trastuzumab) groups, margin of noninferiority, hazard ratio (HR) and its 95% CI for disease-free survival (DFS) and overall survival (OS), and cardiac toxic effects (CHF and low left ventricular ejection fraction [LVEF]) in experimental and control groups.

### IPD Analysis

For IPD analysis, we used the WebPlotDigitizer software^23^ to extract data from published survival curves for both DFS and OS. Data points from survival curves of PHARE^16^ and PERSEPHONE^17^ trials were manually extracted using WebPlotDigitizer because these trials had large numbers of patients and capturing the steps in the curves was difficult in automated data capture. We repeated the process of data extraction from published survival curves to match the reported number of events for each end point in each study as closely as possible. Using this individual-level extracted data and the published numbers at risk, we reconstructed survival curves for DFS and OS for each study using the Stata command ipdfc, published by Wei et al.^22^ The estimated events, HRs, and 95% CIs were compared with those published for each trial. We also estimated the DFS and OS HRs (shorter duration of trastuzumab vs 1 year) and their respective 95% CIs from these data and prepared the corresponding forest plots. We combined the individual-level data of shorter duration and 1-year groups of all included studies except one^11^ and generated the Kaplan-Meier curves for DFS and OS for the entire population. Additionally, we estimated the proportion of DFS and OS for the entire population of 5 trials at each point (ie, 1, 2, 3, 4, and 5 years) using the reconstructed data. For the E 2198 study,^11^ we could not reconstruct the survival curves because the numbers at risk were not provided in the published paper. The detailed methods of data extraction are described in the eAppendix 1 in the Supplement.

### Trial-Level Analysis

For trial-level analysis, we used the published DFS and OS estimates of the randomized populations and relevant subgroups from all 6 eligible RCTs. For each study, we extracted the HRs and their corresponding 95% CIs and obtained log rank observed minus expected events and log rank variance statistics, using the methods described by Tierney et al.^24^ We pooled HR estimates using the fixed-effects model, based on Mantel-Haenszel approach, while a random-effects model was used in subgroup analyses because of heterogeneity among studies. For subgroup analyses, the DFS HRs of the experimental vs control groups were extracted for the following subgroups: age (≤50 and >50 years), stage (I, II, and III), hormone receptor status (estrogen receptor–positive and estrogen receptor–negative], lymph node status (node negative [N0], 1-3 node positive [N1-N2], and ≥4 node positive [N3]), and timing of trastuzumab (concurrent and sequential). We also estimated the number of events for subgroup forest plots for studies in which events were not reported by subgroup and treatment group.^12,13,14,16^ Furthermore, we grouped the shorter duration RCTs according to the duration of trastuzumab (9-12 weeks and 6 months, respectively) and compared both with the results in their respective 1-year groups. Cardiac toxic effects were defined as either symptomatic CHF or asymptomatic decline in LVEF seen on cardiac imaging (by 2-dimensional echocardiography or multigated acquisition scan) or cardiac death. Cardiac evaluation was done at baseline and then approximately every 3 months in all studies (eTable 1 in the Supplement).

### Statistical Analysis

The reconstructed IPD and trial-level meta-analyses were designed to assess noninferiority of shorter duration of trastuzumab compared with 1 year of therapy based on the primary outcome measure of DFS. The definition of DFS was consistent across the included trials (eTable 2 in the Supplement). The noninferiority margin was chosen based on the margins in the included RCT and was their median value (1.3; range, 1.15-1.53). A 1-sided DFS *P* < .025 was considered significant for concluding noninferiority of shorter duration compared with 1-year trastuzumab. If the upper limit of the 95% CI of the estimated HR of shorter duration vs 1-year trastuzumab in IPD and trial-level analyses was less than 1.3, then shorter duration treatment would be regarded as noninferior. All statistical analyses were performed using Review Manager version 5.3 (Cochrane Collaboration, Copenhagen, Denmark) and Stata version 14.0 (StataCorp) statistical software.

The methodological quality of eligible RCTs was assessed using the Cochrane Collaboration Risk of Bias Tool, under the 5 following domains: selection bias, performance bias, detection bias, attrition bias, and reporting bias.^25^ We also graded the quality of generated evidence based on following parameters: risks of bias, imprecision, inconsistency, indirectness, and publication bias.^26^ Publication bias was assessed through funnel plots.

## Results

### Literature Search and Characteristics of Included RCTs

The detailed criteria for exclusion or inclusion of data in the analysis is presented in eFigure 1 in the Supplement. The initial search yielded 365 articles, of which 6 RCTs with 19 publications were included in the final analysis. The duration of adjuvant trastuzumab in experimental arm ranged from 9 to 12 weeks^11,12,13^ to 6 months^14,16,17^; the duration in control groups was 1 year in all included RCTs. The Table lists the important characteristics of RCTs included in the meta-analysis.

**Table.** Description of Randomized Trials Included in the Meta-analysis

Trial	Patients in control group, No.	Patients in experimental group, No.	Primary end point	Secondary end points	Trastuzumab duration in experimental group	Margin of noninferiority, HR	HR (95%CI)		Median follow- up, y
							DFS	OS	
PERSEPHONE^17^	2045; HR+, 1412; N+, 1042; age >50 y, 1388; receiving sequential trastuzumab, 1094	2043; HR+, 1411; N+, 1024; age >50 y, 1366; receiving sequential trastuzumab, 1091	DFS	OS, cardiac dysfunction, and cost- effectiveness	6 mo	<1.32	1.07 (0.93-1.24)	1.14 (0.95-1.37)	5.4
Short-HER^12^	627; HR+, 427; N+, 387	626; HR+, 429; N+, 296	DFS; OS	FR at 2 y and cardiac events	9 wk	<1.29	1.15 (0.91-1.46)	1.06 (0.73-1.55)	5.2
SOLD^13^	1089; HR+, 723; N+, 440	1085; HR+, 711; N+, 438	DFS	DDFS, OS, cardiac DFS, and safety	9 wk	1.3	1.39 (1.12-1.72)	1.36 (0.98-1.89)	5.2
PHARE^16^	1690; HR+, 1021; N+, 763; age >50 y, 1091; receiving sequential trastuzumab, 729	1690; HR+, 1040; N+, 759; age >50 y, 1096; receiving sequential trastuzumab, 747	DFS	OS and cardiac safety	6 mo	1.15	1.08 (0.93-1.25)	1.13 (0.92-1.39)	7.5
HORG^14^	241; HR+, 156; N+, 180	240; HR+, 165; N+, 200	DFS	OS and toxic effects	6 mo	1.53	1.58 (0.86-2.10)	1.45 (0.57-3.67)	4.2
E 2198^11^	112; HR+, 69	115; HR+ 71	Cardiac toxic effects	DFS and OS	12 wk	NA	1.3 (0.8-2.1)	1.4 (0.74-2.54)	6.4

Abbreviations: DDFS, distant disease–free survival; DFS, disease-free survival; FR, failure rate; HR, hazard ratio; HR+, hormone receptor positive; N+, node positive; NA, not applicable; OS, overall survival.

### Risk of Bias

Assessment of risk of bias in included studies is presented in eFigure 2 in the Supplement. The quality of evidence was graded high for DFS and OS but was graded moderate for cardiac toxic effects because of differences in the definition in the included trials (eTable 3 in the Supplement).

### IPD Survival Analysis

**Figure 1.**
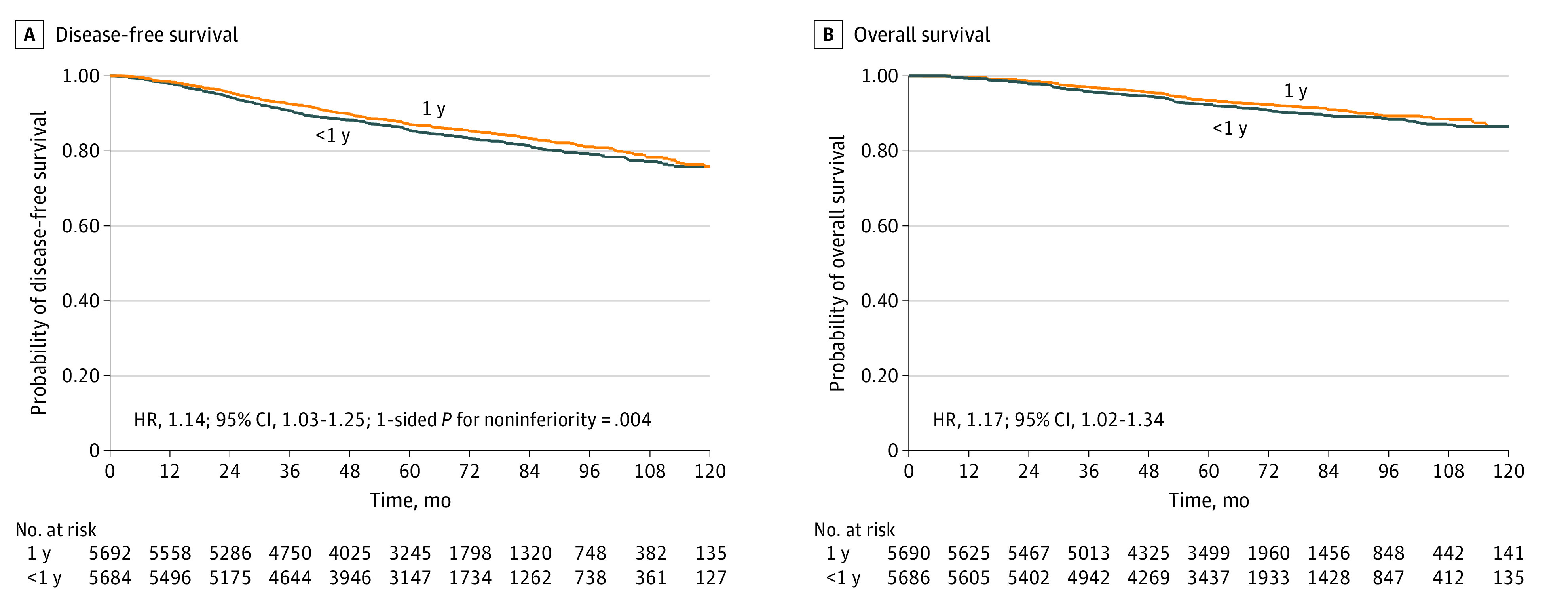
Reconstructed Survival Curves for Individual Patient Data From 5 Randomized Clinical Trials Comparing Shorter Duration vs 1 Year of Trastuzumab A, For patients receiving 1 year of trastuzumab, disease-free survival was 87.12% (95% CI, 86.15%-88.02%); for those receiving less than 1 year of trastuzumab, disease-free survival was 85.42% (95% CI, 84.41%-86.38%). B, For patients receiving 1 year of trastuzumab, overall survival was 93.46% (95% CI, 92.73%-94.13%); for those receiving less than 1 year of trastuzumab, overall survival was 92.39% (95% CI, 91.61%-93.10%). HR indicates hazard ratio.

**Figure 2.**
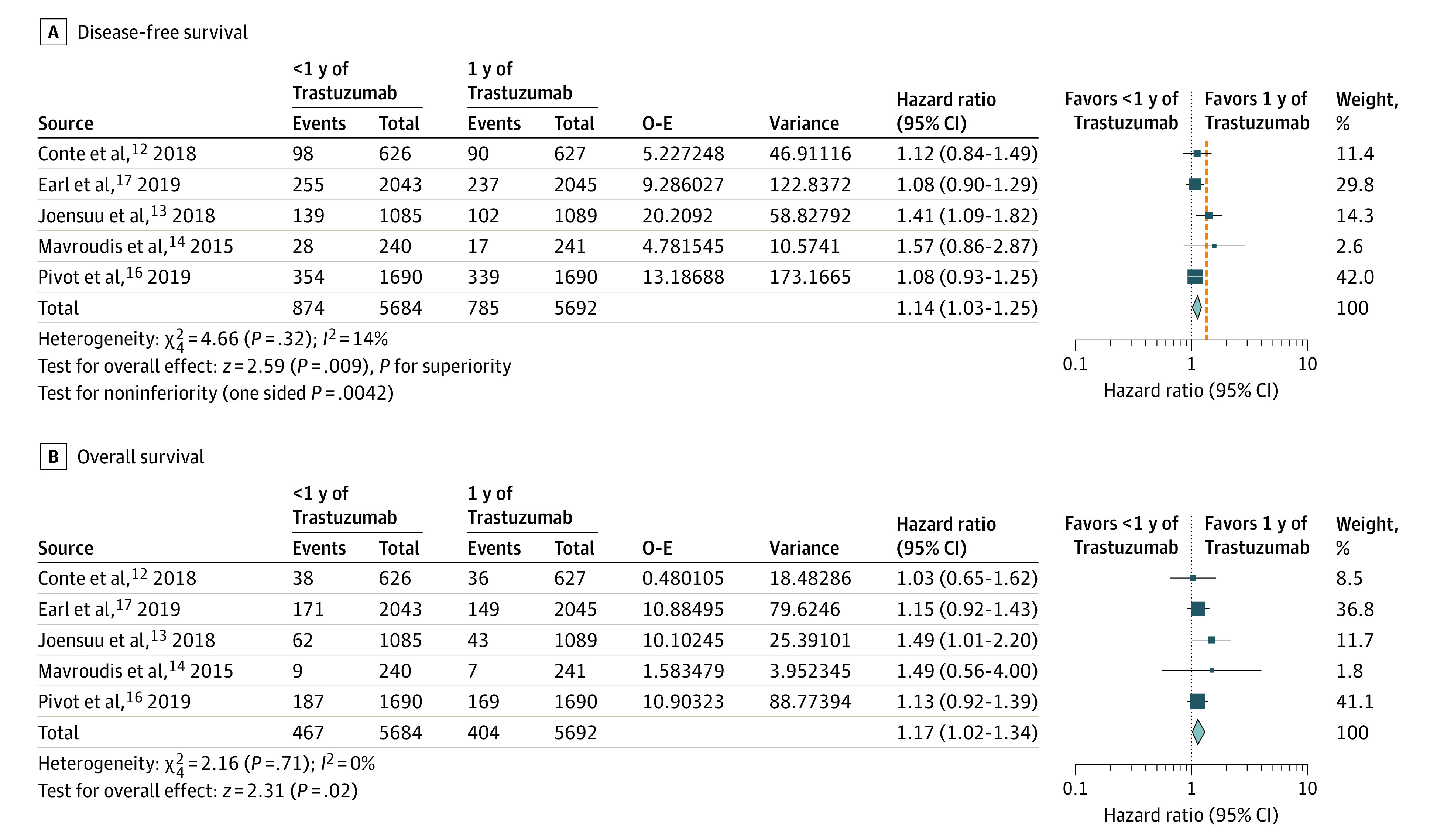
Individual Patient Data Analysis of Disease-Free Survival and Overall Survival Comparing Shorter Duration vs 1 year of Trastuzumab A, Orange line indicates the noninferiority margin. O-E indicates log rank observed minus estimated events.

The IPD analysis included 11 376 patients with 1659 DFS events and 871 deaths from 5 RCTs.^12,13,14,16,17^ The extracted and reported DFS and OS events in each of the 5 included RCTs are shown in eTable 4 in the Supplement and were nearly identical, showing high accuracy of the IPD extraction methodology. The DFS and OS of the combined population of 5 RCTs by duration of trastuzumab are shown in Figure 1. The 5-year DFS was 85.42% (95% CI, 84.41%-86.38%) in the shorter-duration group and 87.12% (95% CI, 86.15%-88.02%) in the 1-year trastuzumab group, with an estimated HR of 1.14 (95% CI, 1.03-1.25; 1-sided *P* for noninferiority = .004) (Figure 2A). The 5-year OS was 92.39% (95% CI, 91.61%-93.10%) in the shorter duration group compared with 93.46% (95% CI, 92.73%-94.13%) in the 1-year trastuzumab group, with an estimated HR of 1.17 (95% CI, 1.02–1.34) (Figure 2B). The DFS and OS estimates at 1, 2, 3, 4, and 5 years, reconstructed from extracted IDP, are shown in eTable 5 and eTable 6 in the Supplement, respectively, for each trial and for the meta-analyzed population by treatment group.

### Trial-Level Survival Analysis

The trial-level analysis included 11 603 patients, 1760 DFS events, and 930 deaths from 6 RCTs^11,12,13,14,16,17^ (eFigure 3 in the Supplement). For shorter duration vs 1 year of trastuzumab, the HR for DFS was 1.15 (95% CI, 1.04-1.26; 1-sided *P* for noninferiority = .002), and the HR for OS was 1.17 (95% CI, 1.03-1.33). These results closely matched those obtained from extracted IPD mentioned previously. When analyzed to test superiority, 1-year administration of trastuzumab resulted in significantly better DFS (HR, 0.87; 95% CI, 0.80-0.96) and OS (HR, 0.86; 95% CI, 0.75-0.97) compared with shorter durations (eFigure 4 in the Supplement).

**Figure 3.**
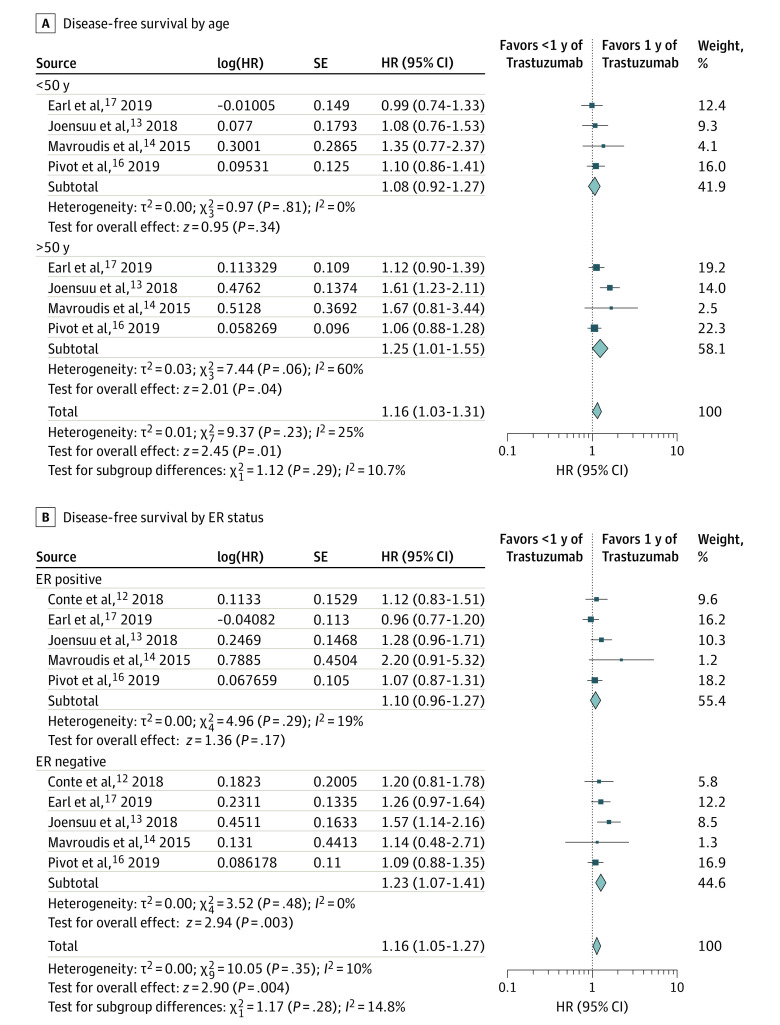
Hazard Ratio (HR) Plots for Disease-Free Survival by Age and Estrogen Receptor Status The number of events in subgroups are based on data in eTable 7 in the Supplement.

**Figure 4.**
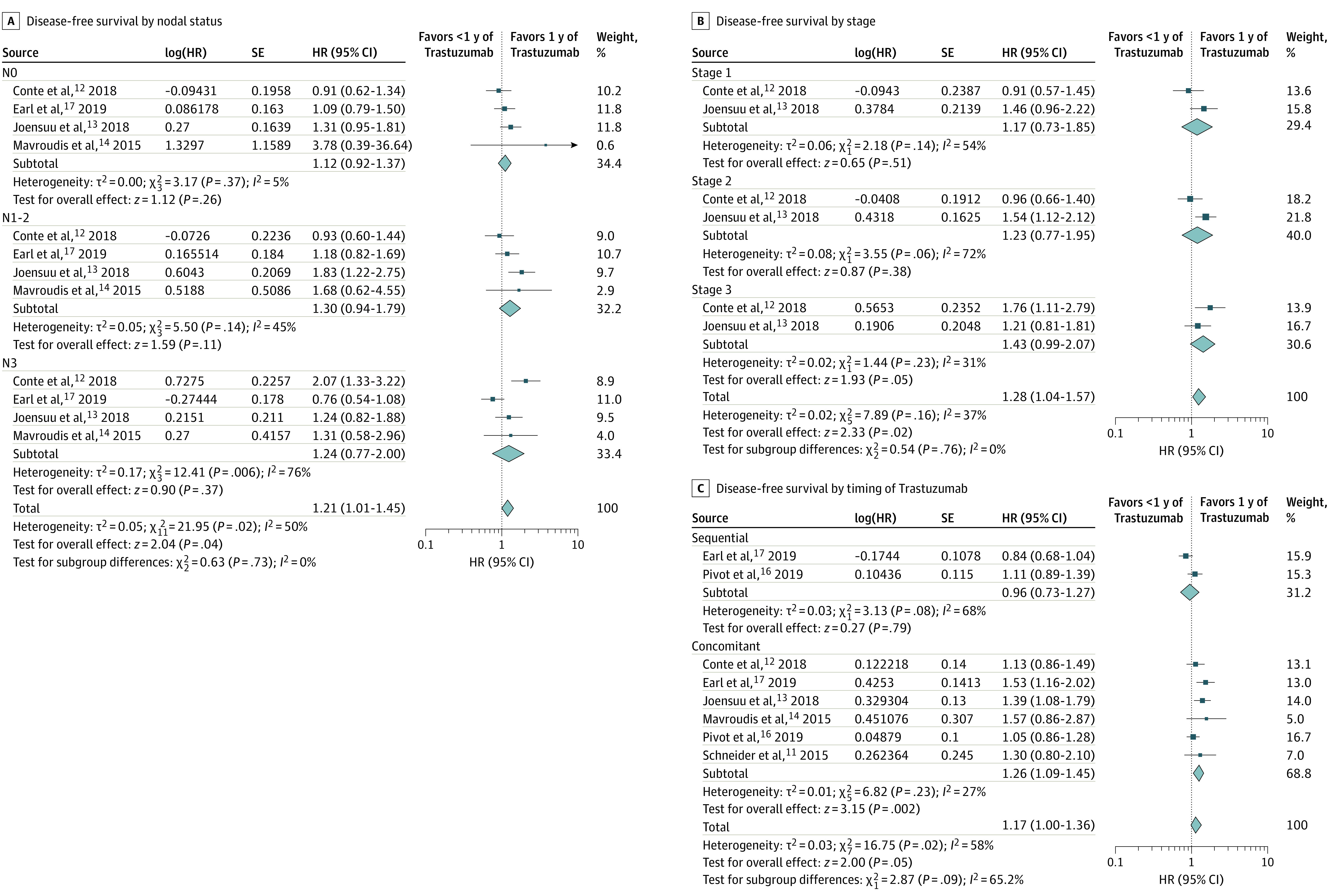
Hazard Ratio (HR) Plots for Disease-Free Survival by Nodal Status, Stage, and Timing of Trastuzumab Administration The number of events in subgroups are based on data in eTable 7 in the Supplement.

Using pooled analyses of published HRs, we analyzed the efficacy (DFS) of shorter duration vs 1 year of trastuzumab in subgroups defined by age (4 trials),^13,14,16,17^ estrogen receptor status (5 trials),^12,13,14,16,17^ lymph node status (4 trials),^12,13,14,17^stage (2 trials)^12,13^ and concomitant vs sequential administration of trastuzumab (2 trials)^16,17^ (Figure 3 and Figure 4; eTable 7 in the Supplement). The test of interaction between subgroups defined by these factors and the association of trastuzumab duration with outcomes was not significant for any factor. However, the lower bound of the 95% CI of the point estimates of HRs for DFS (shorter duration vs 1 year of trastuzumab) did not cross the line of unity, favoring 1 year of trastuzumab, in patients older than 50 years (HR, 1.25; 95% CI, 1.01-1.55), those with estrogen receptor–negative tumors (HR, 1.23; 95% CI, 1.07-1.41), and those who received concomitant trastuzumab administration (HR, 1.26; 95% CI, 1.09–1.45; *P* for superiority = .002). The lower bound of the 95% CI of the point estimates of HRs for DFS (shorter duration vs 1 year of trastuzumab) crossed the line of unity in patients younger than 50 years (HR, 1.08; 95% CI, 0.92-1.27), with estrogen receptor–positive tumors (HR, 1.10; 95% CI, 0.96-1.27), N0 disease (HR, 1.12; 95% CI, 0.92-1.37), N1 to N3 disease (HR, 1.30; 95% CI, 0.94-1.70), stage I disease (HR, 1.17; 95% CI, 0.73-1.85), and stage II disease (HR, 1.23; 95% CI, 0.77-1.95), and those who received sequential trastuzumab administration (HR, 0.96; 95% CI, 0.73-1.27, *P* for superiority = .79). Of note, the interaction between DFS and the timing of trastuzumab treatment (concomitant or sequential) was not statistically significant (Figure 4C).

In the 3 trials that used 9 to 12 weeks of trastuzumab in their experimental group,^11,12,13^ the HRs for DFS and OS (vs 1 year trastuzumab) were 1.27 (95% CI, 1.07-1.51) and 1.25 (95% CI, 0.96-1.63), respectively, while in the 3 trials that used trastuzumab for 6 months in their experimental group,^14,16,17^ the HRs for DFS and OS (vs 1 year trastuzumab) were 1.10 (95% CI, 0.99-1.23) and 1.14 (95% CI, 0.99-1.32), respectively (eFigure 5 in the Supplement).

### Cardiac Toxic Effects

The proportion of patients developing CHF was lower among those receiving shorter duration of trastuzumab compared with those receiving 1 year (3.9% vs 6.9%; RR 0.53; 95% CI, 0.38-0.74; *P* < .001) (eFigure 6 in the Supplement). The proportion of patients developing asymptomatic LVEF decline was also lower among those receiving shorter duration compared with those receiving 1-year trastuzumab (5% vs 7%; RR 0.71; 95% CI, 0.50-1.00; *P* = .049) (eFigure 6 in the Supplement).

## Discussion

Our meta-analysis showed that trastuzumab durations of less than 1 year duration have noninferior DFS compared with 1-year administration of this drug using a noninferiority margin of 1.3 for the upper limit of the 95% CI of the HR. The noninferiority was shown using both extracted IPD from published survival curves and pooled analysis of published HRs, with very similar point estimates and confidence intervals. Furthermore, the small absolute differences in 3-year and 5-year DFS and OS between the shorter duration and 1-year trastuzumab groups suggest that the therapeutic effect of this drug lies along a continuum of duration, with the major fraction of benefit possibly achieved with shorter durations of as long as 6 months. Of course, this assumes an implicit comparison with the pivotal trials that included a no trastuzumab group in their designs. However, it is worth noting that the point estimates of DFS and OS and corresponding absolute survival proportions were numerically in favor of 1-year duration.

Because the absolute differences are small, it is of interest to identify subgroups for whom shorter durations might suffice. Our analysis failed to statistically identify such groups, perhaps because of inadequate power for some interactions. It is likely that the small differences between 1-year and shorter duration trastuzumab in the overall *ERBB2*-positive population are a mean of large as well as nonexistent differences in distinct biological groups, which derive large and no benefit from extended duration trastuzumab, respectively. Our subgroup analysis suggested that in patients with estrogen receptor–positive or N0 disease, 1 year of trastuzumab may add little benefit in terms of DFS. The absolute differences in DFS and OS within various subgroups can only be elucidated in an analysis that has access to IPD within these subgroups. Our analysis confirmed that a shorter duration of trastuzumab was associated with fewer cardiac toxic effects, both in terms of CHF and LVEF decline. However, it is worth mentioning that despite the higher cardiac toxic effects, longer duration of trastuzumab was associated with lower all-cause mortality compared with shorter durations. This is likely a reflection of the reversibility of trastuzumab-associated cardiac toxic effects.

Although adjuvant trastuzumab has been the standard of care for *ERBB2*-positive early breast cancer since 2006, its optimum duration has been uncertain. In particular, it has been a matter of interest to know whether durations less than 1 year would preserve the therapeutic effect of 1 year of trastuzumab while lowering the cardiac toxic effects and cost of treatment. The pivotal trials that proved the efficacy of adjuvant trastuzumab overwhelmingly used this drug for 1 year. Therefore, the burden of proof has been on shorter durations and justifiably so. This has meant that RCTs studying the duration of trastuzumab have been conducted according to noninferiority designs with resulting implications for the interpretation of results. Of the 6 included RCTs, 5 failed to prove noninferiority of shorter durations while 1 proved noninferiority of 6 months of adjuvant trastuzumab compared with its 1-year administration. To our knowledge, this is the only meta-analysis that has preserved the noninferiority interpretation of its constituent trials. Although it is sometimes statistically acceptable to interpret superiority based on results of individual noninferiority trials,^27^ a number of conditions need to be satisfied, and it may be most appropriate to preserve the noninferiority interpretation in a meta-analysis. For example, a previous trial-level meta-analysis^20^ reported a DFS HR for less than 1 year of trastuzumab vs 1 year of trastuzumab of 1.13 (95% CI, 1.03-1.25) and concluded superiority of 1 year of trastuzumab. We obtained an almost identical DFS HR of 1.14 (95% CI, 1.03-1.25) and have concluded noninferiority of less than 1 year trastuzumab. Because the trials included in both meta-analyses have been explicitly planned to prove the noninferiority of shorter durations of trastuzumab, we believe that our interpretation is statistically and thematically robust. However, when analyzed to test superiority, 1-year administration of trastuzumab resulted in significantly better DFS (HR, 0.87; 95% CI, 0.80-0.96) and OS (HR, 0.86; 95% CI, 0.75-0.97) compared with shorter durations.

The noninferiority margin is primarily based on clinical acceptability. The range of noninferiority margins in the constituent trials of our meta-analysis was 1.15 to 1.53, reflecting the range of opinions about acceptable margins of noninferiority. To be objective, we took the median within this range, corresponding to an absolute difference in 5-year DFS of 3.5%, given the control group DFS of 87.12% in our analysis.

There are several clinical implications of our analysis. Overall, it suggests that shorter durations of trastuzumab may be noninferior to 1-year duration in patients with *ERBB2*-positive early breast cancer. Of note, our analyses of 3 trials that used 9 to 12 weeks of trastuzumab and 3 other trials that used 6 months suggest more favorable DFS and OS hazard ratios in the latter set, indicating that abbreviating trastuzumab duration to 6 months may be a more appropriate strategy. The small absolute differences in 5-year DFS and OS between shorter durations and 1 year of trastuzumab suggest that the former may be appropriate in situations of resource constraints or toxic effects. Our results also suggest that shorter durations provide clinically acceptable results in patients with low risk of relapse, such as those with estrogen receptor–positive or N0 disease. This could be considered when increased access is being attempted in currently underserved populations.

### Strengths and Limitations

There are several strengths of our meta-analysis. We have used the most recent publication of trials with updated data. We have used a recently described method to extract individual patient events from published Kaplan-Meier curves and reconstructed the DFS and OS curves by treatment group for each trial (eAppendix 2 in the Supplement) and the entire meta-analyzed population, and we reported an IPD meta-analysis for the main end points of DFS and OS. We provided detailed DFS and OS proportions at clinically relevant time points, enabling physicians to counsel patients regarding absolute detriment or benefit with shorter duration vs 1 year of trastuzumab. By performing a trial-level meta-analysis in addition to IPD analysis, we have attempted to reduce uncertainties regarding either result.

There are some limitations of our analysis, mainly related to the inherent characteristics of the included RCTs. The included trials have differences in study design, variable noninferiority margins, varying durations of trastuzumab in the experimental arm, different chemotherapeutic regimens and schedules, different timing of randomization, and HRs based on variable follow-up durations. An important methodological consideration in this analysis is the timing of randomization, which was prior to starting systemic therapy in trials by Schneider et al,^11^ Joensuu et al,^13^ and Mavroudis et al,^14^ after completion of chemotherapy in the trial by Pivot et al,^16^ and just prior to the 10th trastuzumab cycle in the trial by Earl et al.^17^ The variable timing could have introduced bias by excluding patients who were rapidly relapsing or who were treatment intolerant in the latter 2 trials.^16,17^ These factors could potentially affect the combinability of the study populations. However, there was no significant statistical heterogeneity between the included trials. Subgroup analysis was possible for DFS but not for OS. Furthermore, other clinical end points, such as the site of recurrence and breast cancer specific mortality, could not be evaluated owing to a lack of data.

## Conclusions

The results of this meta-analysis suggest that shorter duration of trastuzumab is noninferior to its 1-year administration with respect to DFS with fewer cardiac toxic effects. The absolute survival differences between the 2 groups are small, and shorter durations could be therapeutically appropriate in situations of toxic effects or resource constraints, especially among patients with clinically low-risk disease.

## Notes

Supplement. **eAppendix 1.** Supplementary Methods **eAppendix 2.** Reconstructed Survival Curves for Each Trial **eFigure 1.** Study Flowchart **eFigure 2.** Risk of Bias in Included Trials **eFigure 3.** Disease-Free Survival and Overall Survival Comparing Shorter Duration vs 1 Year of Trastuzumab Using Published Estimates **eFigure 4.** Disease-Free Survival and Overall Survival Comparing 1 Year vs Shorter Duration of Trastuzumab Using Published Estimates **eFigure 5.** Disease-Free Survival and Overall Survival Comparing Shorter (6 months or 9-12 weeks) Duration vs 1 year of Trastuzumab Using Published Estimates **eFigure 6.** Analysis of Congestive Heart Failure and Decrease in Left Ventricle Ejection Fraction Comparing Shorter Duration vs 1 Year of Trastuzumab Based on Published Estimates **eTable 1.** Frequency of Cardiac Monitoring in Included Trials **eTable 2.** Definition of Disease-Free Survival in Included Trials **eTable 3.** Quality of Evidence **eTable 4.** Estimated and Reported Events and Hazard Ratios, by Trial and Treatment Group **eTable 5.** Estimated Disease-Free Survival at Various Points Using Individual Patient Data From 5 RCTs **eTable 6.** Estimated Overall Survival at Various Points Using Individual Patient Data From 5 RCTs **eTable 7.** Estimated Events for the Subgroups in Each Trial
