# Supplementary material for: Evaluation of 1-Year vs Shorter Durations of Adjuvant Trastuzumab Among Patients With Early Breast Cancer: An Individual Participant Data and Trial-Level Meta-analysis
Source: JAMA Netw Open. 2020 Aug 24;3(8):e2011777. doi: 10.1001/jamanetworkopen.2020.11777 (PMC7445596; doi:10.1001/jamanetworkopen.2020.11777)
Supplement: Supplement. — eAppendix 1. Supplementary Methods eAppendix 2. Reconstructed Survival Curves for Each Trial eFigure 1. Study Flowchart eFigure 2. Risk of Bias in Included Trials eFigure 3. Disease-Free Survival and Overall Survival Comparing Shorter Duration vs 1 Year of Trastuzumab Using Published Estimates eFigure 4. Disease-Free Survival and Overall Survival Comparing 1 Year vs Shorter Duration of Trastuzumab Using Published Estimates eFigure 5. Disease-Free Survival and Overall Survival Comparing Shorter (6 months or 9-12 weeks) Duration vs 1 year of Trastuzumab Using Published Estimates eFigure 6. Analysis of Congestive Heart Failure and Decrease in Left Ventricle Ejection Fraction Comparing Shorter Duration vs 1 Year of Trastuzumab Based on Published Estimates eTable 1. Frequency of Cardiac Monitoring in Included Trials eTable 2. Definition of Disease-Free Survival in Included Trials eTable 3. Quality of Evidence eTable 4. Estimated and Reported Events and Hazard Ratios, by Trial and Treatment Group eTable 5. Estimated Disease-Free Survival at Various Points Using Individual Patient Data From 5 RCTs eTable 6. Estimated Overall Survival at Various Points Using Individual Patient Data From 5 RCTs eTable 7. Estimated Events for the Subgroups in Each Trial [file jamanetwopen-3-e2011777-s001.pdf]

Supplementary Online Content

Gulia S, Kannan S, Badwe R, Gupta S. Evaluation of 1-year vs shorter durations of adjuvant trastuzumab among patients with early breast cancer: an individual participant data and trial-level meta-analysis. *JAMA Netw Open*. 2020;3(8):e2011777. doi:10.1001/jamanetworkopen.2020.11777

eAppendix 1. Supplementary Methods
eAppendix 2. Reconstructed Survival Curves for Each Trial
eFigure 1. Study Flowchart
eFigure 2. Risk of Bias in Included Trials
eFigure 3. Disease-Free Survival and Overall Survival Comparing Shorter Duration vs 1 Year of Trastuzumab Using Published Estimates
eFigure 4. Disease-Free Survival and Overall Survival Comparing 1 Year vs Shorter Duration of Trastuzumab Using Published Estimates
eFigure 5. Disease-Free Survival and Overall Survival Comparing Shorter (6 months or 9-12 weeks) Duration vs 1 year of Trastuzumab Using Published Estimates
eFigure 6. Analysis of Congestive Heart Failure and Decrease in Left Ventricle Ejection Fraction Comparing Shorter Duration vs 1 Year of Trastuzumab Based on Published Estimates
eTable 1. Frequency of Cardiac Monitoring in Included Trials
eTable 2. Definition of Disease-Free Survival in Included Trials
eTable 3. Quality of Evidence
eTable 4. Estimated and Reported Events and Hazard Ratios, by Trial and Treatment Group
eTable 5. Estimated Disease-Free Survival at Various Points Using Individual Patient Data From 5 RCTs
eTable 6. Estimated Overall Survival at Various Points Using Individual Patient Data From 5 RCTs
eTable 7. Estimated Events for the Subgroups in Each Trial

This supplementary material has been provided by the authors to give readers additional information about their work.

© 2020 Gulia S et al. *JAMA Network Open.*

eAppendix 1. Supplementary Methods

A. Detailed methodology of individual patient data extraction and reconstruction of survival curves

WebPlotDigitizer software[25] was used to extract data from the published Kaplan Meier survival curves for both DFS and OS. Data points from survival curves of PERSEPHONE and PHARE trials were extracted manually using WEB Plot digitizer because these trials had large number of patients and capturing the steps in the curves were difficult in automated data capture. The process of extraction of data from published survival curves was repeated, to match, as closely as possible, the reported number of events for each endpoint in each study.

Using this extracted data and the published numbers at risk; we reconstructed Kaplan Meier DFS and OS survival curves for each study using the STATA command ipdfc, published by Wei et al. [22] For one study by Schneider et al, [11] we could not reconstruct the survival curves, as the number at risk was not provided in the published paper.

The forest plot for DFS and OS were obtained using the extracted data of 5 RCT. Individual patient data was combined for all studies except one [11]and Kaplan Meier curve (DFS and OS) by treatment group (duration of trastuzumab) were generated for the combined population of these 5 studies. Additionally, we also estimated the proportions of patients surviving and events, at each time point (1-year, 2year, 3 year, 4 year and 5 year) using the individual patient data with estimation of HR and 90% or 95% CI. The HR and the confidence interval calculated from extracted individual patient data were compared with the reported rates.

B. Statistical Methods Used to Estimate Events Among Subgroups

	< 1 year	1 year	Events/total	HR (95% CI)
Subgroup	Events/total	Events/total		
< 50	a/n_{11}	b/n_{12}	$a+b/N_{10}$	Reported
>=50	c/n_{21}	d/n_{22}	$c+d/N_{20}$	Reported
Total	$a+c/N_{01}$	$b+d/N_{02}$	$(a+b+c+d)/N$	

Where a,b,c,d was not reported but a+b, c+d, a+c and b+dwas reported. However, all studies have reported n_{11}, n_{12}, n_{21}, and $n_{22,}$ as well asN_{01}, N_{02}, N_{10} and N_{20}.

For the above mentioned data structure the following method was used to determine a,b,c,d. Expected frequencies for a,b,c,d were calculated based on marginal totals similar to the calculation of expected cell frequencies in chi-square test.

Observed events were calculated using the following formula from Tierney etal.[26]

$$\mathrm{HR} = \left[\frac{\textit{Observed events research / logrank Expected events research}}{\textit{Observed events control / logrank Expected events control}}\right]$$

The reported hazard ratio and the expected events obtained using the above method was substituted in the above formula to calculate the observed events.

The observed events obtained using the above method was reported in the subgroup forest plots. However, these events were not used as inputs to calculate the HR and 95% CI for the random effects model for subgroup analysis.

eAppendix 2. Reconstructed Survival Curves for Each Trial

1. Pivot X, et al[16] (PHARE trial)

1.1: Extracted DFS events from PHARE trial

```
      failure _d:  event_ipd
analysis time _t:  t_ipd
```

Time	Beg. Total	Fail	Survivor Function	Std. Error	[95% Conf. Int.]	
1 year						
12	1626	50	0.9703	0.0041	0.9609	0.9774
24	1550	51	0.9396	0.0058	0.9270	0.9500
36	1471	53	0.9071	0.0071	0.8921	0.9201
48	1378	50	0.8758	0.0081	0.8588	0.8908
60	1216	35	0.8525	0.0088	0.8342	0.8689
72	1105	25	0.8343	0.0094	0.8150	0.8517
84	922	27	0.8122	0.0100	0.7916	0.8310
96	611	22	0.7890	0.0109	0.7667	0.8095
108	293	18	0.7558	0.0131	0.7290	0.7803
120	45	8	0.7169	0.0195	0.6767	0.7531
132	39	0	0.7169	0.0195	0.6767	0.7531
< 1 year						
12	1599	71	0.9577	0.0049	0.9469	0.9663
24	1494	72	0.9141	0.0069	0.8995	0.9266
36	1415	57	0.8789	0.0080	0.8621	0.8937
48	1325	38	0.8549	0.0087	0.8369	0.8711
60	1154	29	0.8352	0.0092	0.8162	0.8524
72	1057	28	0.8142	0.0098	0.7940	0.8326
84	886	23	0.7948	0.0104	0.7736	0.8143
96	607	18	0.7762	0.0111	0.7536	0.7969
108	285	13	0.7539	0.0124	0.7287	0.7772
120	53	5	0.7370	0.0143	0.7079	0.7638
132	47	0	0.7370	0.0143	0.7079	0.7638

Note: Survivor function is calculated over full data and evaluated at indicated times; it is not calculated from aggregates shown at left.

Events in one-year arm – 339

Events in less than one-year arm – 354

© 2020 Gulia S et al. *JAMA Network Open.*

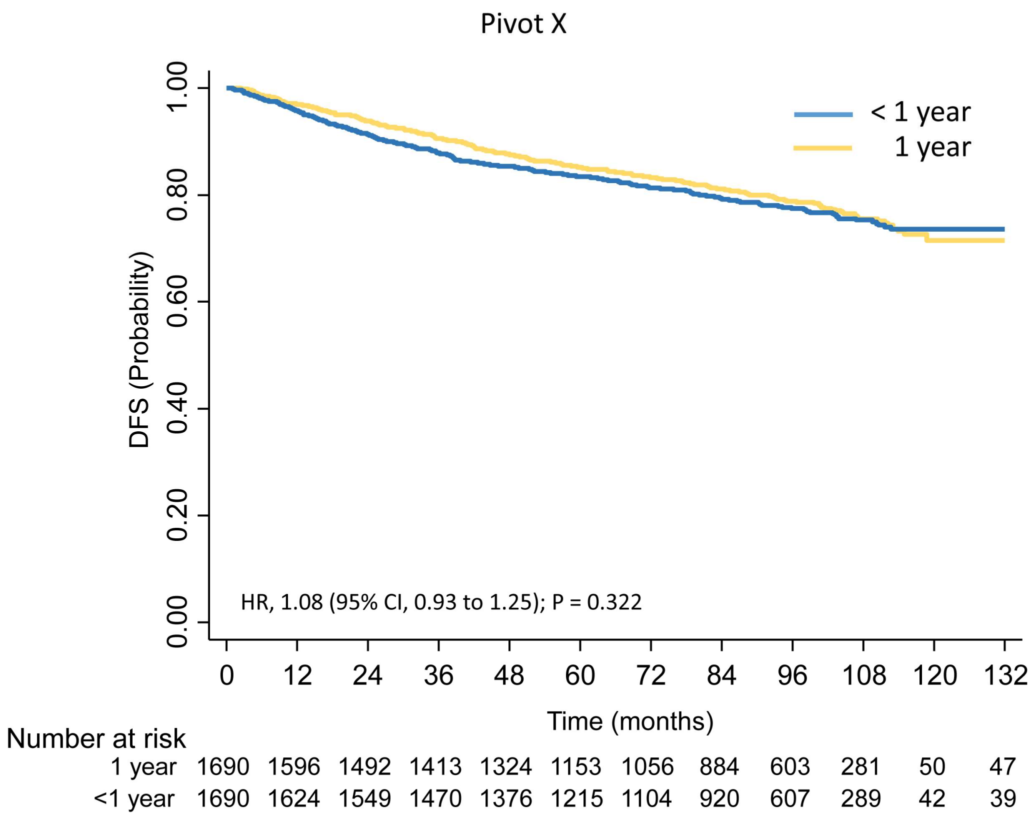

Reconstructed disease-free survival curve (DFS) of PHARE trial

© 2020 Gulia S et al. *JAMA Network Open.*

1.1.1: Extracted OS events from PHARE trial

```
        failure _d:  event_ipd
   analysis time _t:  t_ipd
```

Time	Beg. Total	Fail	Survivor Function	Std. Error	[95% Conf. Int.]	
1 year						
12	1675	3	0.9982	0.0010	0.9945	0.9994
24	1631	20	0.9862	0.0029	0.9793	0.9908
36	1576	28	0.9691	0.0043	0.9595	0.9764
48	1494	31	0.9497	0.0054	0.9379	0.9593
60	1338	21	0.9357	0.0061	0.9225	0.9467
72	1218	17	0.9233	0.0067	0.9090	0.9355
84	1031	19	0.9077	0.0075	0.8918	0.9214
96	699	18	0.8888	0.0086	0.8707	0.9046
108	354	5	0.8793	0.0095	0.8593	0.8967
120	54	7	0.8489	0.0148	0.8172	0.8755
< 1 year						
12	1662	15	0.9911	0.0023	0.9852	0.9946
24	1594	33	0.9711	0.0041	0.9618	0.9781
36	1540	32	0.9514	0.0053	0.9399	0.9608
48	1464	25	0.9358	0.0061	0.9227	0.9466
60	1294	23	0.9203	0.0068	0.9059	0.9326
72	1192	18	0.9070	0.0074	0.8914	0.9204
84	1010	22	0.8890	0.0082	0.8719	0.9040
96	699	7	0.8812	0.0086	0.8631	0.8970
108	335	10	0.8640	0.0101	0.8430	0.8825
120	57	2	0.8583	0.0108	0.8357	0.8781

Note: Survivor function is calculated over full data and evaluated at indicated times; it is not calculated from aggregates shown at left.

Events in one-year arm – 169

Events in less than one-year arm – 187

© 2020 Gulia S et al. *JAMA Network Open.*

Pivot X

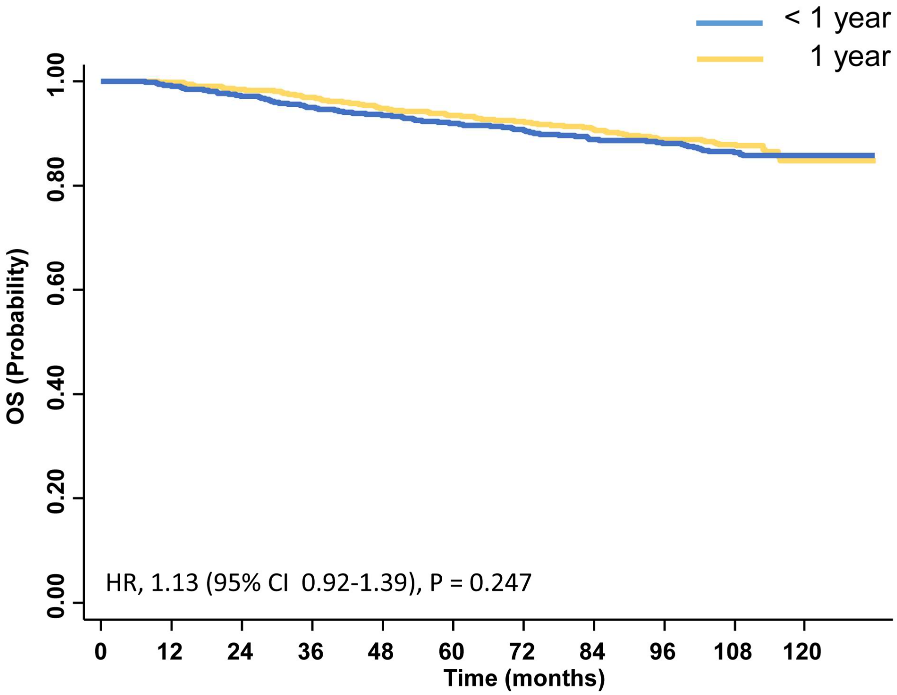

Number at risk

**1 year**	**1690**	**1660**	**1591**	**1539**	**1463**	**1292**	**1191**	**1008**	**698**	**331**	**54**
**<1 year**	**1690**	**1674**	**1630**	**1575**	**1493**	**1336**	**1217**	**1029**	**695**	**350**	**50**

Reconstructed overall survival curve (OS) of PHARE trial

© 2020 Gulia S et al. *JAMA Network Open.*

2. Joensuu H et al [13] (SOLD trial)

2.1: Extracted DFS events from SOLD trial

```
        failure _d:  event_ipd
  analysis time _t:  t_ipd
```

Time	Beg. Total	Fail	Survivor Function	Std. Error	[95% Conf. Int.]	
1 year						
12	1075	9	0.9917	0.0028	0.9841	0.9957
24	1047	23	0.9704	0.0052	0.9584	0.9790
36	901	22	0.9490	0.0068	0.9339	0.9607
48	751	21	0.9250	0.0084	0.9067	0.9398
60	579	12	0.9082	0.0096	0.8875	0.9252
72	397	6	0.8974	0.0104	0.8750	0.9160
84	245	5	0.8815	0.0125	0.8546	0.9037
96	87	4	0.8584	0.0169	0.8215	0.8882
108	87	0	0.8584	0.0169	0.8215	0.8882
< 1 year						
12	1060	12	0.9888	0.0032	0.9804	0.9936
24	1014	31	0.9596	0.0060	0.9460	0.9699
36	863	33	0.9268	0.0081	0.9092	0.9411
48	716	23	0.9001	0.0096	0.8796	0.9173
60	546	13	0.8820	0.0106	0.8594	0.9012
72	383	11	0.8615	0.0121	0.8358	0.8834
84	236	6	0.8453	0.0136	0.8165	0.8699
96	82	10	0.7997	0.0193	0.7586	0.8346
108	82	0	0.7997	0.0193	0.7586	0.8346

Note: Survivor function is calculated over full data and evaluated at indicated times; it is not calculated from aggregates shown at left.

Events in one-year arm- 102

Events in less than one-year arm - 139

© 2020 Gulia S et al. *JAMA Network Open.*

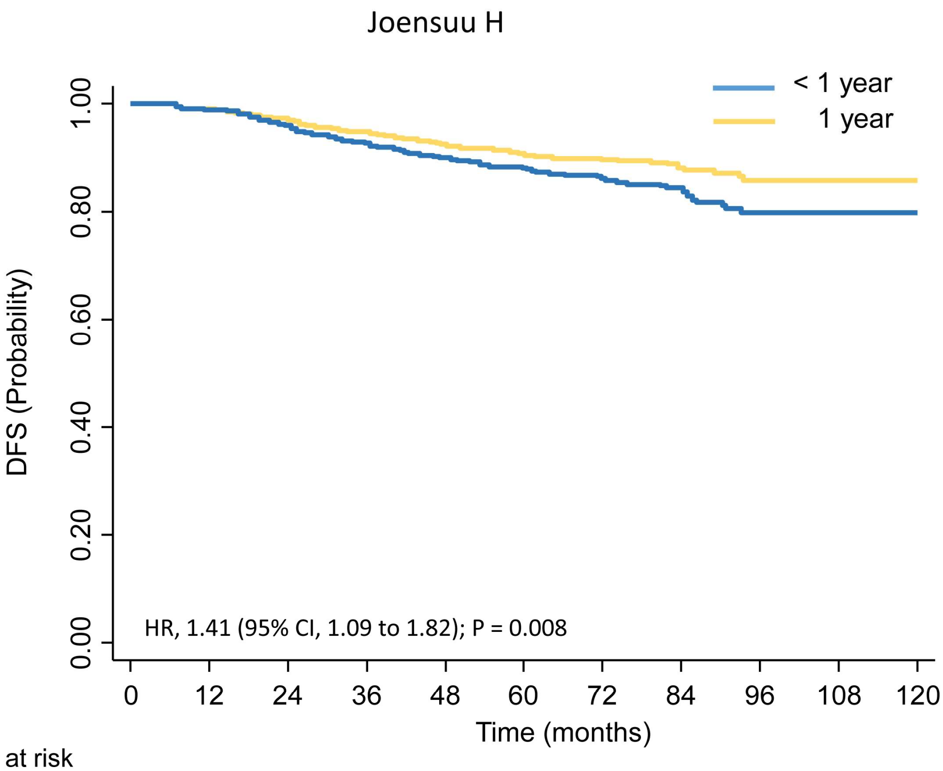

Number at risk

1 year	1085	1060	1014	860	707	545	381	228	76	76	76
<1 year	1089	1074	1047	889	742	566	394	245	87	82	82

Reconstructed disease-free survival curve (DFS) of SOLD trial

© 2020 Gulia S et al. *JAMA Network Open.*

2.1.1: Extracted OS events from SOLD trial

Time	Beg. Total	Fail	Survivor Function	Std. Error	[95% Conf. Int.]	
1 year						
12	1088	0	1.0000	.	.	.
24	1081	6	0.9945	0.0023	0.9877	0.9975
36	947	10	0.9847	0.0038	0.9752	0.9906
48	796	12	0.9712	0.0054	0.9585	0.9801
60	615	9	0.9586	0.0068	0.9431	0.9700
72	433	3	0.9536	0.0073	0.9368	0.9660
84	279	0	0.9536	0.0073	0.9368	0.9660
96	96	3	0.9357	0.0126	0.9057	0.9563
108	96	0	0.9357	0.0126	0.9057	0.9563
< 1 year						
12	1076	0	1.0000	.	.	.
24	1052	18	0.9831	0.0039	0.9734	0.9893
36	914	8	0.9750	0.0048	0.9635	0.9829
48	770	9	0.9645	0.0059	0.9508	0.9744
60	593	13	0.9469	0.0076	0.9299	0.9599
72	419	11	0.9268	0.0096	0.9056	0.9434
84	256	1	0.9243	0.0099	0.9024	0.9414
96	97	2	0.9102	0.0141	0.8783	0.9341
108	88	0	0.9102	0.0141	0.8783	0.9341

Note: Survivor function is calculated over full data and evaluated at indicated times; it is not calculated from aggregates shown at left.

Events in one-year arm – 43

Events in less than one-year arm - 62

© 2020 Gulia S et al. *JAMA Network Open.*

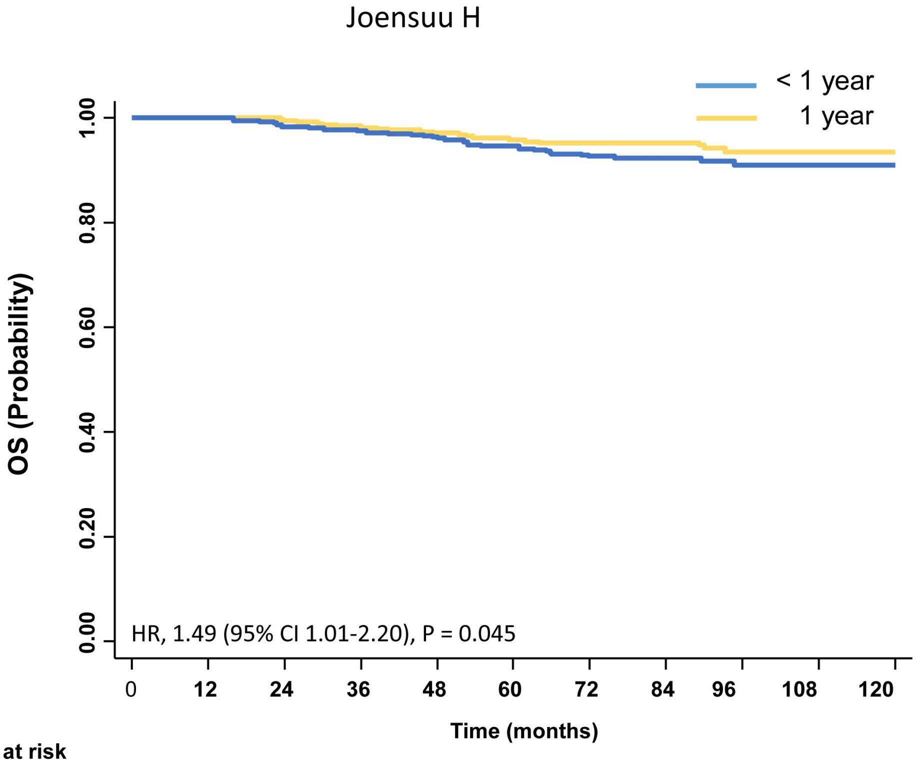

Number at risk

**1 year**	**1085**	**1075**	**1047**	**907**	**761**	**585**	**418**	**247**	**88**	**81**	**81**
**<1 year**	**1089**	**1087**	**1078**	**937**	**786**	**605**	**421**	**263**	**87**	**87**	**87**

Reconstructed overall survival curve (OS) of SOLD trial

© 2020 Gulia S et al. *JAMA Network Open.*

3. Earl H etal[17] (PERSEPHONE trial)

3.1: Extracted DFS events from PERSEPHONE trial

```
        failure _d:  event_ipd
  analysis time _t:  t_ipd
```

Time	Beg. Total	Fail	Survivor Function	Std. Error	[95% Conf. Int.]	
1 year						
0	0	0	1.0000	.	.	.
1	2015	16	0.9921	0.0020	0.9872	0.9952
2	1890	70	0.9571	0.0045	0.9473	0.9652
3	1670	61	0.9251	0.0060	0.9125	0.9359
4	1307	44	0.8982	0.0070	0.8835	0.9111
5	1012	46	0.8627	0.0085	0.8451	0.8784
< 1 year						
0	0	0	1.0000	.	.	.
1	2009	18	0.9911	0.0021	0.9860	0.9944
2	1879	72	0.9550	0.0046	0.9450	0.9633
3	1651	77	0.9142	0.0064	0.9008	0.9258
4	1319	35	0.8941	0.0071	0.8794	0.9072
5	1016	53	0.8528	0.0087	0.8348	0.8691

Events in one-year arm- 237

Events in less than one-year arm - 255

© 2020 Gulia S et al. *JAMA Network Open.*

Earl H

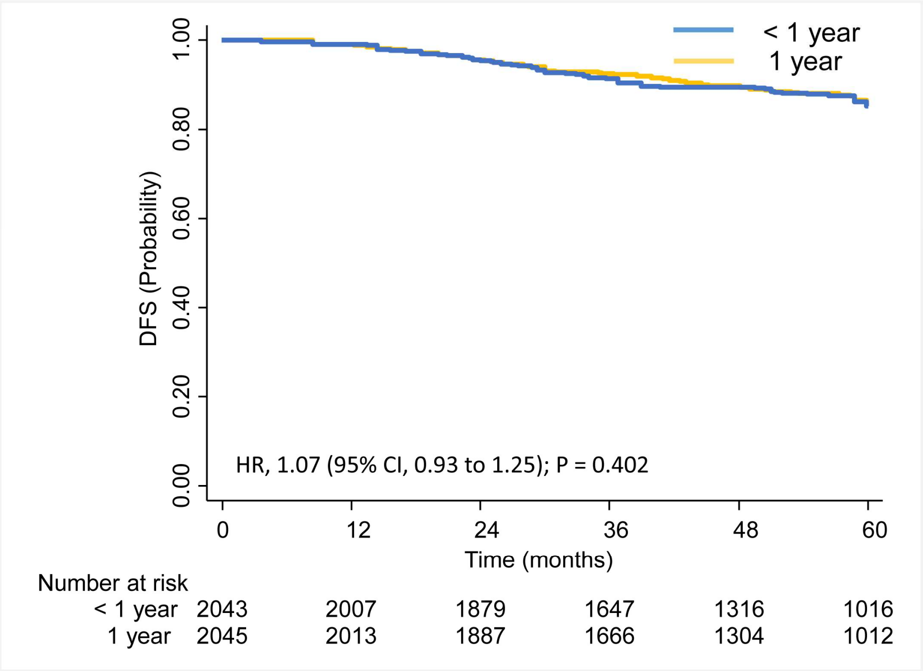

Reconstructed disease-free survival curve (DFS) of PERSEPHONE trial

© 2020 Gulia S et al. *JAMA Network Open.*

3.1.1: Extracted OS events from PERSEPHONE trial

```
         failure _d:  event_ipd
   analysis time _t:  t_ipd
```

Time	Beg. Total	Fail	Survivor Function	Std. Error	[95% Conf. Int.]	
1 year						
0	0	0	1.0000	.	.	.
1	2020	16	0.9921	0.0020	0.9872	0.9952
2	1942	24	0.9802	0.0031	0.9731	0.9854
3	1737	38	0.9602	0.0044	0.9506	0.9680
4	1396	20	0.9479	0.0052	0.9368	0.9571
5	1064	51	0.9096	0.0072	0.8943	0.9227
< 1 year						
0	0	0	1.0000	.	.	.
1	2015	18	0.9912	0.0021	0.9860	0.9944
2	1945	32	0.9752	0.0035	0.9674	0.9811
3	1735	59	0.9442	0.0052	0.9331	0.9535
4	1406	11	0.9379	0.0055	0.9262	0.9478
5	1062	51	0.9006	0.0074	0.8851	0.9141

Events in one-year arm- 149

Events in less than one-year arm – 171

© 2020 Gulia S et al. *JAMA Network Open.*

Earl H

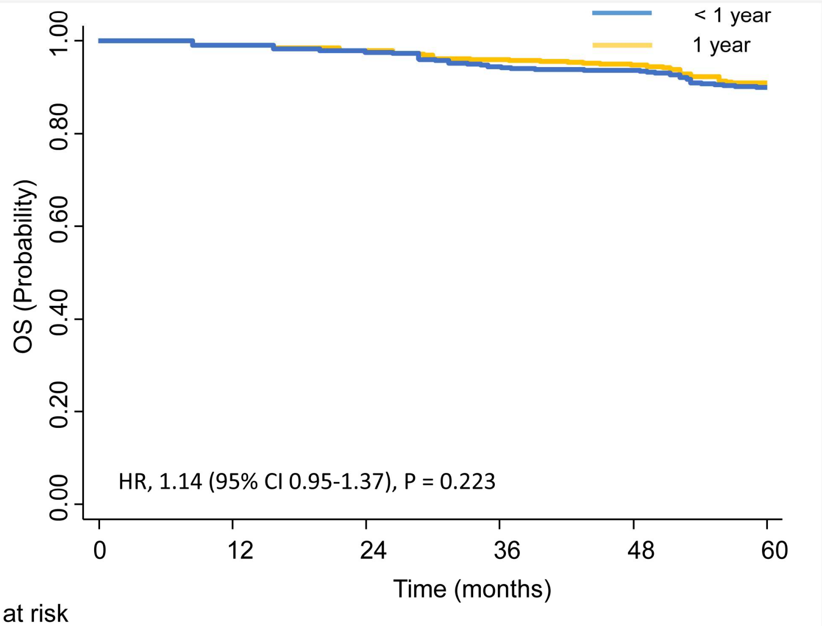

Number at risk

1 year	2043	2014	1937	1731	1405	1062
<1 year	2045	2019	1939	1732	1395	1064

Reconstructed overall survival curve (OS) of PERSEPHONE trial

© 2020 Gulia S et al. *JAMA Network Open.*

4. Conte PF et al [12] (SHORT – HER trial)

4.1: Extracted DFS events from SHORT- HER trial

Time	Beg. Total	Fail	Survivor Function	Std. Error	[95% Conf. Int.]	
1 year						
12	610	10	0.9839	0.0051	0.9703	0.9913
24	594	14	0.9612	0.0078	0.9426	0.9738
36	568	25	0.9206	0.0109	0.8962	0.9394
48	490	11	0.9012	0.0121	0.8745	0.9224
60	381	14	0.8739	0.0138	0.8441	0.8984
72	254	10	0.8450	0.0161	0.8103	0.8738
84	144	4	0.8291	0.0177	0.7912	0.8608
96	43	2	0.8159	0.0197	0.7735	0.8511
< 1 year						
12	604	10	0.9838	0.0051	0.9700	0.9912
24	580	24	0.9444	0.0093	0.9231	0.9600
36	556	20	0.9116	0.0115	0.8861	0.9316
48	481	15	0.8852	0.0130	0.8568	0.9082
60	358	15	0.8538	0.0149	0.8218	0.8805
72	257	8	0.8322	0.0164	0.7972	0.8616
84	134	4	0.8154	0.0181	0.7768	0.8480
96	46	2	0.7999	0.0209	0.7553	0.8373

Note: Survivor function is calculated over full data and evaluated at indicated times; it is not calculated from aggregates shown at left.

Events in one-year arm- 90

Events in less than one-year arm - 98

© 2020 Gulia S et al. *JAMA Network Open.*

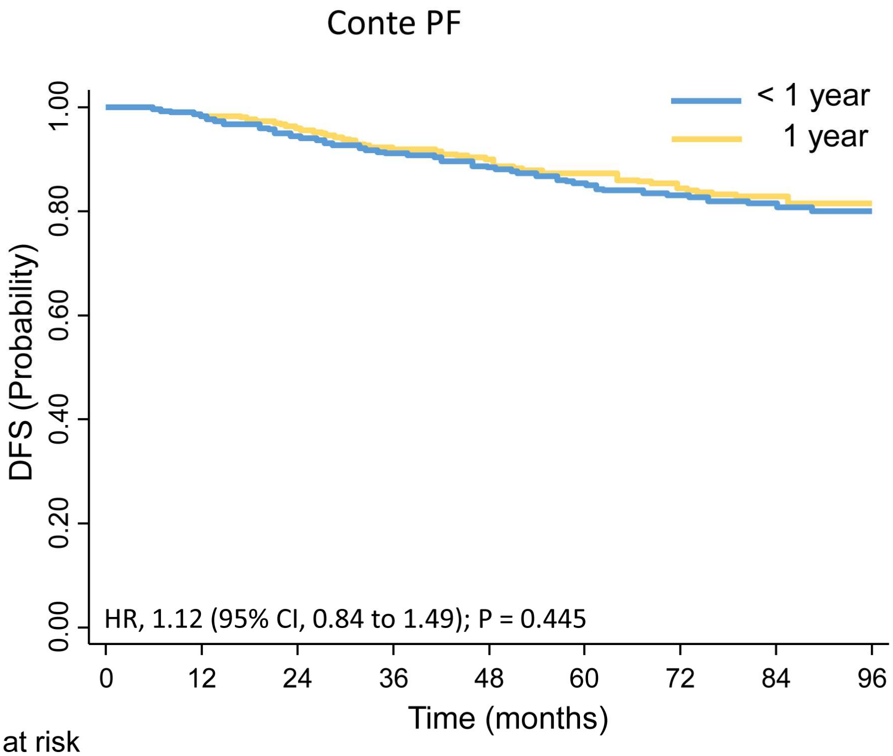

Number at risk

1 year	626	602	576	554	480	351	245	120	46
<1 year	627	608	592	566	488	374	251	132	43

Reconstructed disease-free survival curve (DFS) of Short-HER trial

© 2020 Gulia S et al. *JAMA Network Open.*

3.1.1: Extracted OS events from SHORT-HER trial

```
         failure _d:  event_ipd
   analysis time _t:  t_ipd
```

Time	Beg. Total	Fail	Survivor Function	Std. Error	[95% Conf. Int.]	
1 year						
12	611	0	1.0000	.	.	.
24	604	6	0.9901	0.0040	0.9782	0.9956
36	592	7	0.9786	0.0059	0.9634	0.9875
48	520	7	0.9663	0.0074	0.9482	0.9781
60	410	7	0.9512	0.0092	0.9294	0.9664
72	271	5	0.9365	0.0112	0.9105	0.9552
84	140	3	0.9219	0.0139	0.8895	0.9451
96	54	1	0.9147	0.0156	0.8785	0.9405
< 1 year						
12	617	1	0.9984	0.0016	0.9886	0.9998
24	613	2	0.9951	0.0028	0.9850	0.9984
36	603	6	0.9853	0.0049	0.9720	0.9923
48	525	15	0.9593	0.0082	0.9398	0.9725
60	419	4	0.9511	0.0091	0.9298	0.9660
72	275	4	0.9407	0.0104	0.9166	0.9579
84	153	4	0.9247	0.0129	0.8949	0.9463
96	54	2	0.9078	0.0176	0.8665	0.9368

Note: Survivor function is calculated over full data and evaluated at indicated times; it is not calculated from aggregates shown at left.

Events in one-year arm- 36

Events in less than one-year arm - 38

© 2020 Gulia S et al. *JAMA Network Open.*

Conte PF

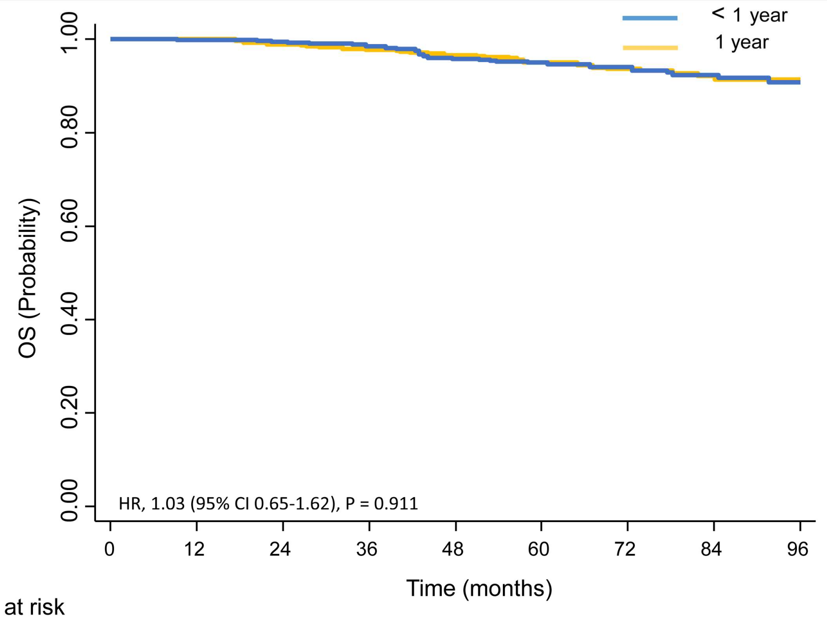

Number at risk

1 year	627	616	612	601	524	412	271	149	50
<1 year	626	610	602	591	516	399	258	129	50

Reconstructed overall survival curve (OS) of SHORT-HER trial

© 2020 Gulia S et al. *JAMA Network Open.*

5. Mavroudis D et al[14] (HORG trial)

5.1: Extracted DFS events from HORG trial

Time	Beg. Total	Fail	Survivor Function	Std. Error	[95% Conf. Int.]	
1 year						
12	240	0	1.0000	.	.	.
24	213	4	0.9824	0.0087	0.9538	0.9934
36	161	5	0.9562	0.0144	0.9170	0.9771
48	118	3	0.9363	0.0181	0.8894	0.9637
60	80	4	0.8975	0.0258	0.8335	0.9377
72	50	1	0.8858	0.0280	0.8171	0.9298
84	24	0	0.8858	0.0280	0.8171	0.9298
96	13	0	0.8858	0.0280	0.8171	0.9298
108	13	0	0.8858	0.0280	0.8171	0.9298
< 1 year						
12	232	3	0.9873	0.0073	0.9611	0.9959
24	218	3	0.9742	0.0104	0.9434	0.9883
36	173	8	0.9336	0.0172	0.8902	0.9603
48	123	8	0.8833	0.0238	0.8269	0.9222
60	85	3	0.8568	0.0277	0.7923	0.9024
72	54	3	0.8157	0.0352	0.7345	0.8742
84	32	0	0.8157	0.0352	0.7345	0.8742
96	14	0	0.8157	0.0352	0.7345	0.8742
108	5	0	0.8157	0.0352	0.7345	0.8742

Note: Survivor function is calculated over full data and evaluated at indicated times; it is not calculated from aggregates shown at left.

Events in one-year arm- 17

Events in less than one-year arm – 28

© 2020 Gulia S et al. *JAMA Network Open.*

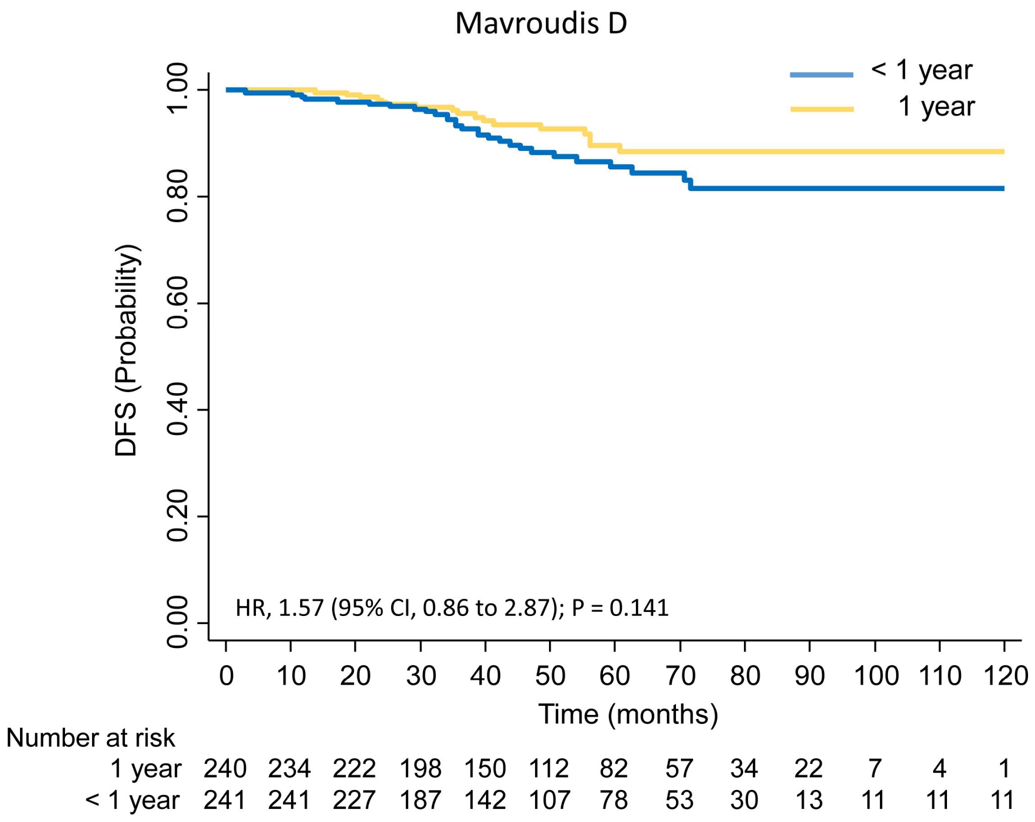

Reconstructed disease-free survival curve (DFS) of HORG trial

© 2020 Gulia S et al. *JAMA Network Open.*

5.1.1: Extracted OS events from HORG trial

```
        failure _d:  event_ipd
  analysis time _t:  t_ipd

              Beg.                             Survivor      Std.
    Time     Total     Fail                    Function      Error     [95% Conf. Int.]
-------------------------------------------------------------------------------
1 year
      12       236        1                     0.9958    0.0042     0.9708    0.9994
      24       219        0                     0.9958    0.0042     0.9708    0.9994
      36       180        2                     0.9858    0.0082     0.9563    0.9954
      48       139        0                     0.9858    0.0082     0.9563    0.9954
      60        97        1                     0.9778    0.0114     0.9400    0.9919
      72        65        1                     0.9642    0.0175     0.9077    0.9864
      84        36        1                     0.9460    0.0249     0.8688    0.9784
      96        17        1                     0.9145    0.0393     0.7952    0.9657
     108         6        0                     0.9145    0.0393     0.7952    0.9657
< 1 year
      12       241        0                     1.0000         .          .         .
      24       222        0                     1.0000         .          .         .
      36       167        3                     0.9829    0.0098     0.9478    0.9944
      48       117        1                     0.9766    0.0116     0.9389    0.9912
      60        87        1                     0.9676    0.0146     0.9225    0.9866
      72        55        3                     0.9298    0.0256     0.8583    0.9659
      84        26        1                     0.9086    0.0326     0.8191    0.9550
      96        13        0                     0.9086    0.0326     0.8191    0.9550
     108        11        0                          .         .          .         .
-------------------------------------------------------------------------------
Note: Survivor function is calculated over full data and evaluated at indicated times; it
      is not calculated from aggregates shown at left.
```

Events in one-year arm- 7

Events in less than one-year arm – 9

© 2020 Gulia S et al. *JAMA Network Open.*

Mavroudis D

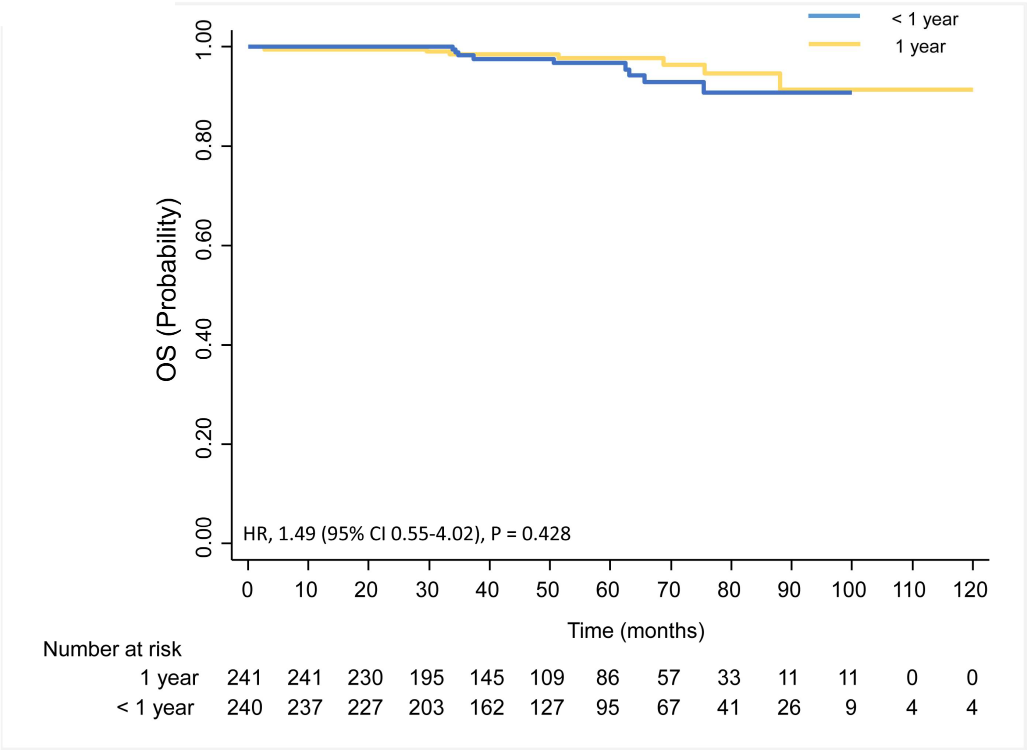

Reconstructed overall survival curve (OS) of HORG trial

© 2020 Gulia S et al. *JAMA Network Open.*

eFigure 1. Study Flowchart

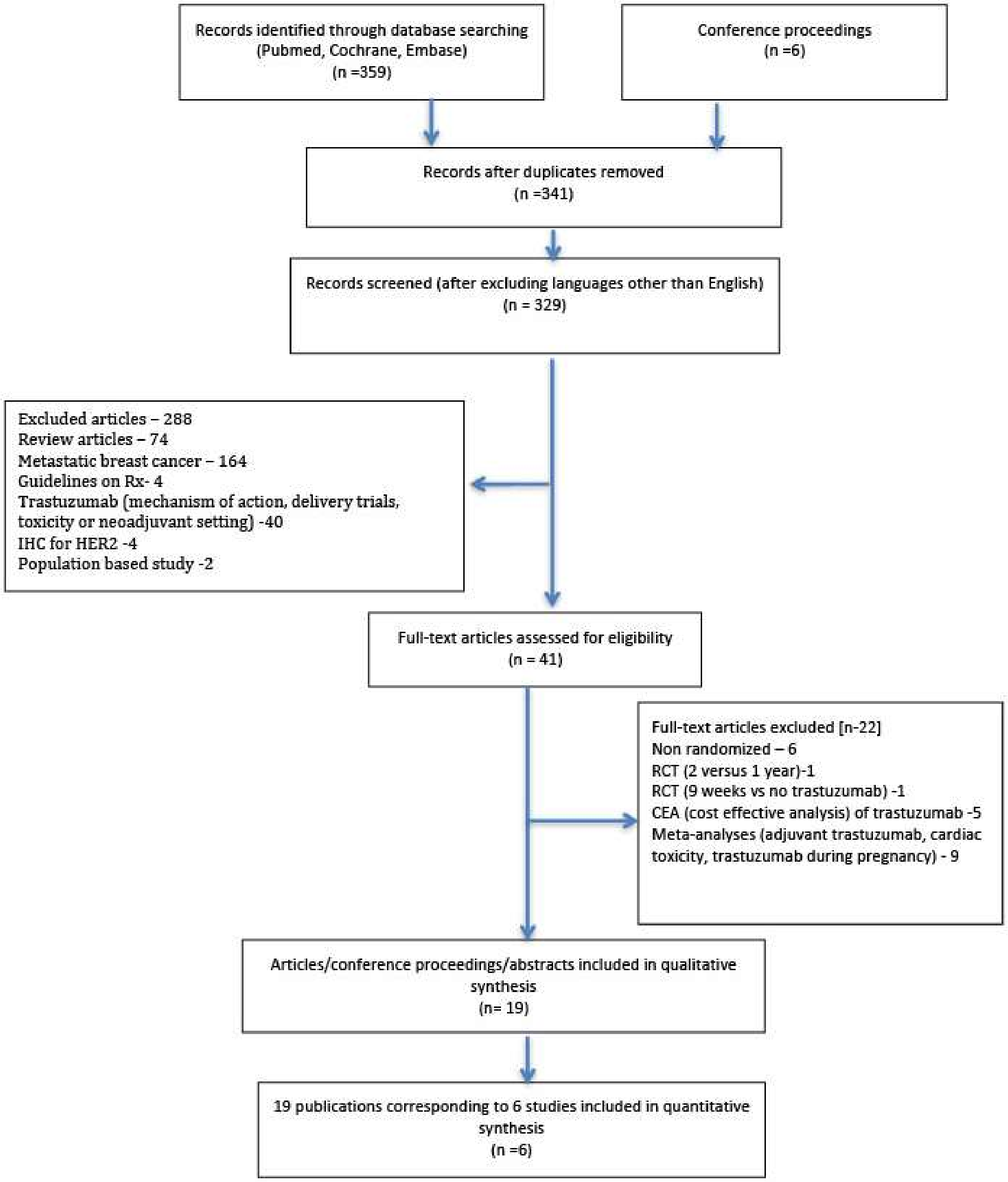

© 2020 Gulia S et al. *JAMA Network Open.*

eFigure 2. Risk of Bias in Included Trials

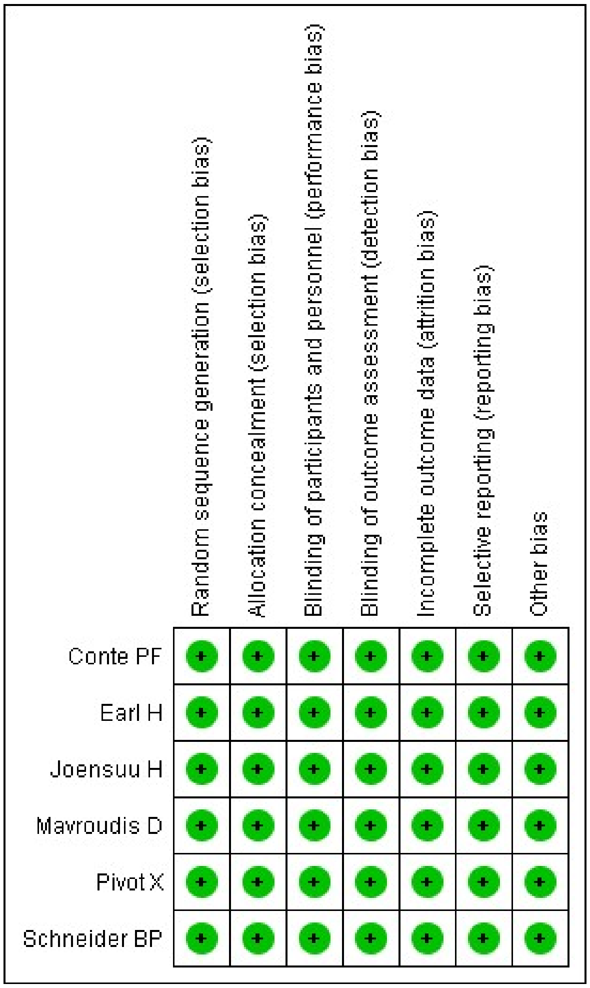

© 2020 Gulia S et al. *JAMA Network Open.*

eFigure 3. Disease-Free Survival and Overall Survival Comparing Shorter Duration vs 1 Year of Trastuzumab Using Published Estimates

A, Disease free Survival comparing shorter duration versus 1 year of trastuzumab using published estimates

e Fig 2(A)

Study or Subgroup	< 1 year Events	< 1 year Total	1 year Events	1 year Total	O-E	Variance	Weight	Hazard Ratio Exp[(O-E) / V], Fixed, 95% CI
Conte PF	105	626	95	627	6.09	49.87	11.1%	1.13 [0.86, 1.49]
Earl H	265	2043	247	2045	8.65	127.84	28.5%	1.07 [0.90, 1.27]
Joensuu H	140	1085	105	1089	19.76	60.00	13.4%	1.39 [1.08, 1.79]
Mavroudis D	28	240	17	241	8.70	19.28	4.3%	1.57 [1.00, 2.45]
Pivot X	359	1690	345	1690	13.52	175.72	39.1%	1.08 [0.93, 1.25]
Schneider BP	25	115	29	112	4.33	16.50	3.7%	1.30 [0.80, 2.11]
**Total (95% CI)**		**5799**		**5804**			**100.0%**	**1.15 [1.04, 1.26]**
Total events	922		838					

Heterogeneity: Chi² = 5.64, df = 5 (P = 0.34); I² = 11%
Test for Superiority: Z = 2.88 (P = 0.004)
Test for Non inferiority (one sided P = 0.002186)

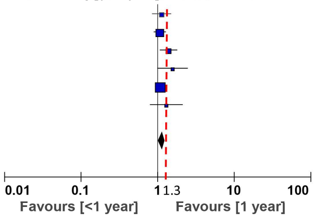

B, Overall survival comparing shorter duration versus 1 year of trastuzumab using published estimates

e Figure 2 (B)

Study	< 1 year Events	< 1 year Total	1 year Events	1 year Total	O-E	Variance	Weight	Hazard Ratio Exp[(O-E) / V], Fixed, 95% CI
Conte PF	40	626	38	627	1.32	19.49	8.4%	1.07 [0.69, 1.67]
Earl H	179	2043	156	2045	10.92	83.35	35.8%	1.14 [0.92, 1.41]
Joensuu H	58	1085	44	1089	7.69	25.02	10.8%	1.36 [0.92, 2.01]
Mavroudis D	8	240	10	241	1.65	4.43	1.9%	1.45 [0.57, 3.68]
Pivot X	186	1690	170	1690	11.03	90.23	38.8%	1.13 [0.92, 1.39]
Schneider BP	18	115	23	112	3.18	10.10	4.3%	1.37 [0.74, 2.54]
**Total (95% CI)**		**5799**		**5804**			**100.0%**	**1.17 [1.03, 1.33]**
Total events	489		441					

Heterogeneity: Chi² = 1.34, df = 5 (P = 0.93); I² = 0%
Test for overall effect: Z = 2.35 (P = 0.02)

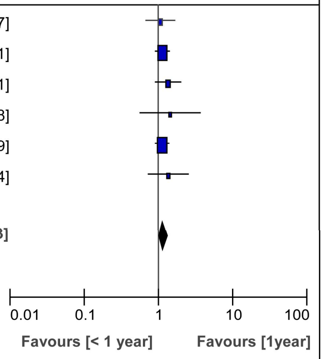

© 2020 Gulia S et al. *JAMA Network Open.*

eFigure 4. Disease-Free Survival and Overall Survival Comparing 1 Year vs Shorter Duration of Trastuzumab Using Published Estimates

A, Disease free Survival comparing 1 year versus shorter duration of trastuzumab using published estimates.

e Fig 5(A)

	<1 year		1 year					Hazard Ratio
**Study or Subgroup**	**Events**	**Total**	**Events**	**Total**	**O-E**	**Variance**	**Weight**	**Exp[(O-E) / V], Fixed, 95% CI**
Conte PF	105	626	95	627	-6.095604	49.875	11.1%	0.88 [0.67, 1.17]
Earl H	265	2043	247	2045	-8.649603	127.8418	28.5%	0.93 [0.79, 1.11]
Joensuu H	140	1085	105	1089	-19.75822	60	13.4%	0.72 [0.56, 0.93]
Mavroudis D	28	240	17	241	-8.696646	19.2798	4.3%	0.64 [0.41, 1.00]
Pivot X	359	1690	345	1690	-13.5238	175.7226	39.1%	0.93 [0.80, 1.07]
Schneider BP	25	115	29	112	-4.328619	16.49851	3.7%	0.77 [0.47, 1.25]
**Total (95% CI)**		**5799**		**5804**			**100.0%**	**0.87 [0.80, 0.96]**
Total events	922		838					

Heterogeneity: Chi² = 5.64, df = 5 (P = 0.34); I² = 11%
Test for overall effect: Z = 2.88 (P = 0.004)

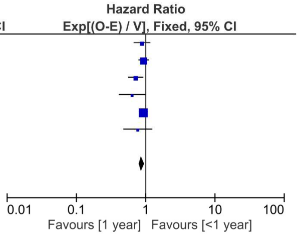

B, Overall survival comparing 1 year versus shorter duration of trastuzumab using published estimates.

e Fig 5(B)

	1 year		<1 year					Hazard Ratio
**Study or Subgroup**	**Events**	**Total**	**Events**	**Total**	**O-E**	**Variance**	**Weight**	**Exp[(O-E) / V], Fixed, 95% CI**
Conte PF	40	626	38	627	-1.318476	19.48718	8.4%	0.93 [0.60, 1.46]
Earl H	179	2043	156	2045	-10.92189	83.35522	35.8%	0.88 [0.71, 1.09]
Joensuu H	58	1085	44	1089	-7.693147	25.01961	10.8%	0.74 [0.50, 1.09]
Mavroudis D	8	240	10	241	-1.64627	4.430655	1.9%	0.69 [0.27, 1.75]
Pivot X	186	1690	170	1690	-11.02727	90.22648	38.8%	0.88 [0.72, 1.09]
Schneider BP	18	115	23	112	-3.180577	10.10314	4.3%	0.73 [0.39, 1.35]
**Total (95% CI)**		**5799**		**5804**			**100.0%**	**0.86 [0.75, 0.97]**
Total events	489		441					

Heterogeneity: Chi² = 1.34, df = 5 (P = 0.93); I² = 0%
Test for overall effect: Z = 2.35 (P = 0.02)

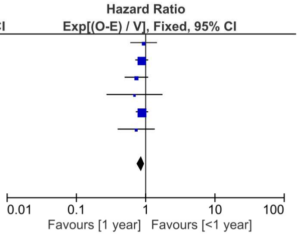

© 2020 Gulia S et al. *JAMA Network Open.*

eFigure 5. Disease-Free Survival and Overall Survival Comparing Shorter (6 months or 9-12 weeks) Duration vs 1 year of Trastuzumab Using Published Estimates

A, Disease-free survival comparing shorter (6 months or 9-12 weeks) duration versus 1 year of trastuzumab using published estimates

e Fig 3 (A)

Study or Subgroup	< 1 year Events	< 1 year Total	1 year Events	1 year Total	O-E	Variance	Weight	Hazard Ratio Exp[(O-E) / V], Fixed, 95% CI
**9.3.1 9 to 12 weeks**								
Conte PF	105	626	95	627	6.095604	49.875	11.1%	1.13 [0.86, 1.49]
Joensuu H	140	1085	105	1089	19.75822	60	13.4%	1.39 [1.08, 1.79]
Schneider BP	25	115	29	112	4.328619	16.49851	3.7%	1.30 [0.80, 2.11]
**Subtotal (95% CI)**		**1826**		**1828**			**28.1%**	**1.27 [1.07, 1.51]**
Total events	270		229					
Heterogeneity: Chi² = 1.18, df = 2 (P = 0.55); I² = 0%								
Test for overall effect: Z = 2.68 (P = 0.007)								
**9.3.2 6 months**								
Earl H	265	2043	247	2045	8.649603	127.8418	28.5%	1.07 [0.90, 1.27]
Mavroudis D	28	240	17	241	8.696646	19.2798	4.3%	1.57 [1.00, 2.45]
Pivot X	359	1690	345	1690	13.5238	175.7226	39.1%	1.08 [0.93, 1.25]
**Subtotal (95% CI)**		**3973**		**3976**			**71.9%**	**1.10 [0.99, 1.23]**
Total events	652		609					
Heterogeneity: Chi² = 2.60, df = 2 (P = 0.27); I² = 23%								
Test for overall effect: Z = 1.72 (P = 0.09)								
**Total (95% CI)**		**5799**		**5804**			**100.0%**	**1.15 [1.04, 1.26]**
Total events	922		838					
Heterogeneity: Chi² = 5.64, df = 5 (P = 0.34); I² = 11%								
Test for overall effect: Z = 2.88 (P = 0.004)								
Test for subgroup differences: Chi² = 1.86, df = 1 (P = 0.17), I² = 46.3%								

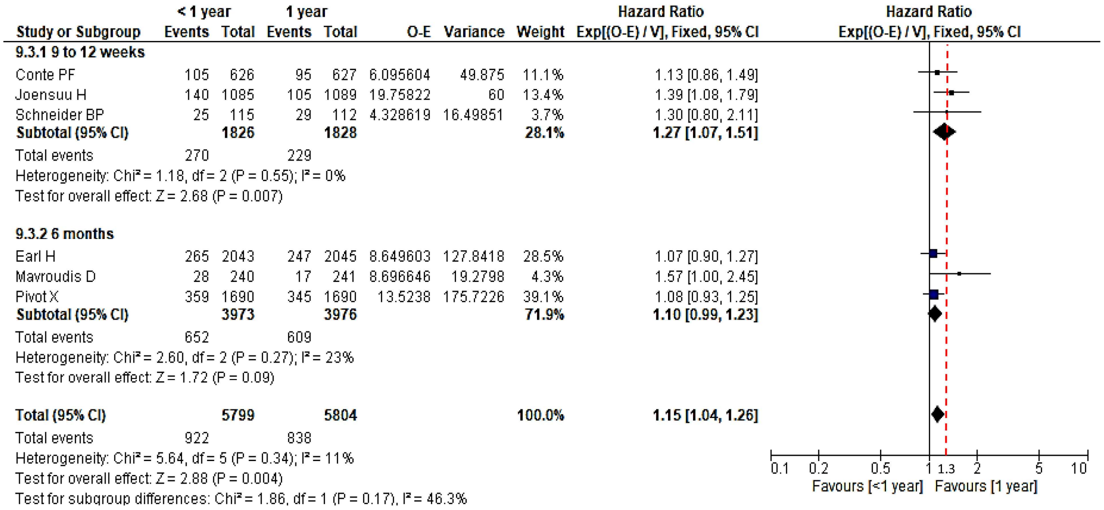

B, Overall survival comparing shorter (6 months or 9-12 weeks) duration versus 1 year of trastuzumab using published estimates

e Figure 3 (B)

Study	< 1 year Events	< 1 year Total	1 year Events	1 year Total	O-E	Variance	Weight	Hazard Ratio Exp[(O-E) / V], Fixed, 95% CI
**9 to 12 weeks**								
Conte PF	40	626	38	627	1.32	19.49	8.4%	1.07 [0.69, 1.67]
Joensuu H	58	1085	44	1089	7.69	25.02	10.8%	1.36 [0.92, 2.01]
Schneider BP	18	115	23	112	3.18	10.10	4.3%	1.37 [0.74, 2.54]
**Subtotal (95% CI)**		**1826**		**1828**			**23.5%**	**1.25 [0.96, 1.63]**
Total events	116		105					
Heterogeneity: Chi² = 0.73, df = 2 (P = 0.69); I² = 0%								
Test for overall effect: Z = 1.65 (P = 0.10)								
**6 months**								
Earl H	179	2043	156	2045	10.92	83.35	35.8%	1.14 [0.92, 1.41]
Mavroudis D	8	240	10	241	1.65	4.43	1.9%	1.45 [0.57, 3.68]
Pivot X	186	1690	170	1690	11.03	90.23	38.8%	1.13 [0.92, 1.39]
**Subtotal (95% CI)**		**3973**		**3976**			**76.5%**	**1.14 [0.99, 1.32]**
Total events	373		336					
Heterogeneity: Chi² = 0.26, df = 2 (P = 0.88); I² = 0%								
Test for overall effect: Z = 1.77 (P = 0.08)								
**Total (95% CI)**		**5799**		**5804**			**100.0%**	**1.17 [1.03, 1.33]**
Total events	489		441					
Heterogeneity: Chi² = 1.34, df = 5 (P = 0.93); I² = 0%								
Test for overall effect: Z = 2.35 (P = 0.02)								
Test for subgroup differences: Chi² = 0.34, df = 1 (P = 0.56), I² = 0%								

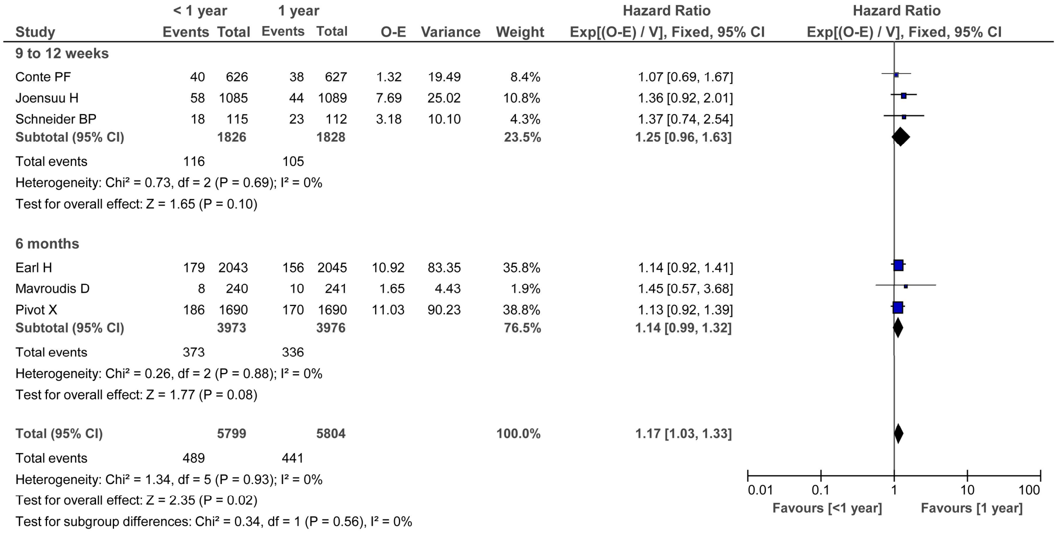

© 2020 Gulia S et al. *JAMA Network Open.*

eFigure 6. Analysis of Congestive Heart Failure and Decrease in Left Ventricle Ejection Fraction Comparing Shorter Duration vs 1 Year of Trastuzumab Based on Published Estimates

A, Analysis of Congestive heart failure comparing shorter duration versus 1 year of trastuzumab based on published estimates.

e Figure 1(A)

	<1 year		1 year			Risk Ratio
**Study**	**Events**	**Total**	**Events**	**Total**	**Weight**	**M-H, Random, 95% CI**
Conte PF	32	626	90	627	26.4%	0.36 [0.24, 0.52]
Earl H	155	1994	224	1968	35.7%	0.68 [0.56, 0.83]
Joensuu H	21	1085	36	1089	20.3%	0.59 [0.34, 1.00]
Pivot X	9	1690	21	1690	12.9%	0.43 [0.20, 0.93]
Schneider BP	3	115	4	112	4.7%	0.73 [0.17, 3.19]
**Total (95% CI)**		**5510**		**5486**	**100.0%**	**0.53 [0.38, 0.74]**
Total events	220		375			

Heterogeneity: Tau² = 0.07; Chi² = 9.43, df = 4 (P = 0.05); I² = 58%
Test for overall effect: Z = 3.71 (P = 0.0002)

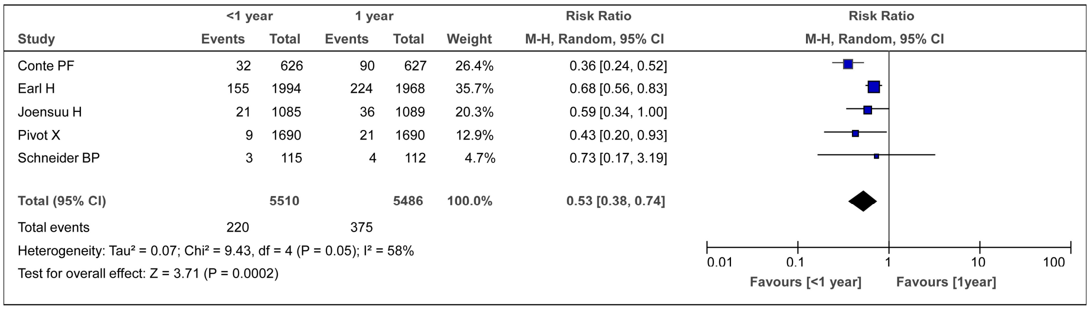

B, Analysis of Decrease in left ventricle ejection fraction comparing shorter duration versus 1 year of trastuzumab based on published estimates.

e Figure1(B)

	<1 year		1 year			Risk Ratio
**Study**	**Events**	**Total**	**Events**	**Total**	**Weight**	**M-H, Random, 95% CI**
Earl H	176	2038	228	2040	46.9%	0.77 [0.64, 0.93]
Joensuu H	1	1085	6	1089	2.5%	0.17 [0.02, 1.39]
Pivot X	58	1690	103	1690	37.4%	0.56 [0.41, 0.77]
Schneider BP	12	112	9	111	13.2%	1.32 [0.58, 3.01]
**Total (95% CI)**		**4925**		**4930**	**100.0%**	**0.71 [0.50, 1.00]**
Total events	247		346			

Heterogeneity: Tau² = 0.06; Chi² = 6.83, df = 3 (P = 0.08); I² = 56%
Test for overall effect: Z = 1.96 (P = 0.05)

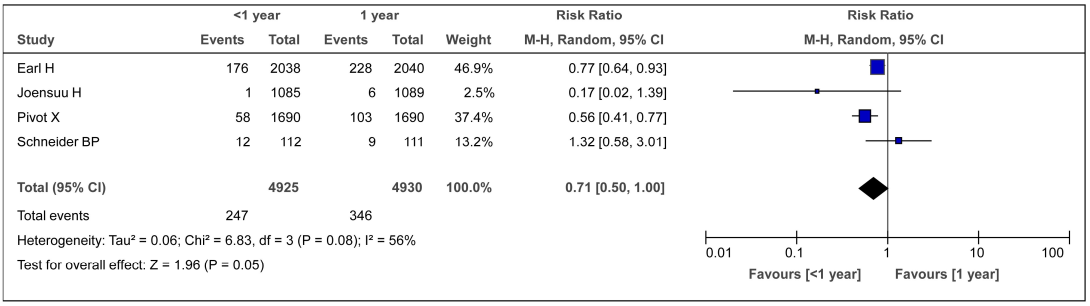

© 2020 Gulia S et al. *JAMA Network Open.*

eTable 1. Frequency of Cardiac Monitoring in Included Trials

Study	Cardiac monitoring
Earl H	Every 3 months initially then every 4 months from 2013 onwards
Joensuu H	Baseline, at study weeks 18, 31, 43, and 61, and 36 months
Pivot X	Every 3 months during the first 2 years and then every 6 months afterwards
Conte PF	At the end of AC/EC, at end of TH then 6, 9, 12, 18 months from randomization, and once every year thereafter
Schneider BP	At baseline, post TH, post AC, 6 months after beginning maintenance trastuzumab; within 1 month of completing; and 1-year post maintenance trastuzumab
Mavroudis D	At 3 months interval

Abbreviations: AC/EC, doxorubicin cyclophosphamide/epirubicin cyclophosphamide; TH, paclitaxel trastuzumab.

© 2020 Gulia S et al. *JAMA Network Open.*

eTable 2. Definition of Disease-Free Survival in Included Trials

Table 6

Study	DFS calculated from	DFS events<br>Local recurrence	Regional recurrence	Distant recurrence	Contralateral breast cancer	Any invasive breast cancer recurrence	Second primary cancer	Death
Earl H[17]	date of diagnostic biopsy				✓	✓		✓
Joensuu H [13]	date of randomization	✓	✓	✓	✓		✓	✓
Pivot X [16]	date of randomization	✓	✓	✓	✓		✓	✓
Conte PF[12]	date of randomization	✓	✓	✓	✓		✓	✓
Schneider BP[11]	date of randomization					✓	✓	✓
Mavroudis D[14]	date of randomization					✓	✓	✓

© 2020 Gulia S et al. *JAMA Network Open.*

eTable 3. Quality of Evidence

(A) : DFS and OS

< 1-year Trastuzumab for Breast Cancer

Patient or population: patients with Breast Cancer
Settings:
Intervention:< 1-year Trastuzumab

Outcomes	Illustrative comparative risks* (95% CI)		Relative effect (95% CI)	No of Participants (studies)	Quality of the evidence (GRADE)	Comments
	Assumed risk	Corresponding risk				
	Control	< 1-year Trastuzumab				
DFS-Extracted data	Study population		HR 1.14 (1.03 to 1.25)	11376 (5 studies)	⊕⊕⊕⊕ high	
	138 per 1000	156 per 1000 (142 to 169)				
	Moderate					
	116 per 1000	131 per 1000 (119 to 143)				
OS-Extracted data	Study population		HR 1.17 (1.02 to 1.34)	11376 (5 studies)	⊕⊕⊕⊕ high	
	71 per 1000	83 per 1000 (72 to 94)				
	Moderate					
	57 per 1000	66 per 1000 (58 to 76)				

*The basis for the assumed risk (e.g. the median control group risk across studies) is provided in footnotes. The corresponding risk (and its 95% confidence interval) is based on the assumed risk in the comparison group and the relative effect of the intervention (and its 95% CI).

CI: Confidence interval; RR: Risk ratio; HR: Hazard ratio;

GRADE Working Group grades of evidence
High quality: Further research is very unlikely to change our confidence in the estimate of effect.
Moderate quality: Further research is likely to have an important impact on our confidence in the estimate of effect and may change the estimate.
Low quality: Further research is very likely to have an important impact on our confidence in the estimate of effect and is likely to change the estimate.
Very low quality: We are very uncertain about the estimate.

© 2020 Gulia S et al. *JAMA Network Open.*

(B) : Cardiac toxicity

Cardiac Toxicity for Breast Cancer						
Patient or population: patients with Breast Cancer<br>Settings:<br>Intervention: Cardiac Toxicity						
Outcomes	Illustrative comparative risks* (95% CI)		Relative effect (95% CI)	No of Participants (studies)	Quality of the evidence (GRADE)	Comments
	Assumed risk	Corresponding risk				
	Control	Cardiac Toxicity				
Cardiac Toxicity	Study population		RR 0.53 (0.38 to 0.74)	10996 (5 studies)	⊕⊕⊕⊖ moderate[1]	
	68 per 1000	36 per 1000 (26 to 51)				
	Moderate					
	36 per 1000	19 per 1000 (14 to 27)				
Low LVF	Study population		RR 0.71 (0.5 to 1)	9855 (4 studies)	⊕⊕⊕⊖ moderate[1]	
	70 per 1000	50 per 1000 (35 to 70)				
	Moderate					
	71 per 1000	50 per 1000 (35 to 71)				
*The basis for the assumed risk (e.g. the median control group risk across studies) is provided in footnotes. The corresponding risk (and its 95% confidence interval) is based on the assumed risk in the comparison group and the relative effect of the intervention (and its 95% CI).<br><br>CI: Confidence interval; RR: Risk ratio;						
GRADE Working Group grades of evidence<br>High quality: Further research is very unlikely to change our confidence in the estimate of effect.<br>Moderate quality: Further research is likely to have an important impact on our confidence in the estimate of effect and may change the estimate.<br>Low quality: Further research is very likely to have an important impact on our confidence in the estimate of effect and is likely to change the estimate.<br>Very low quality: We are very uncertain about the estimate.						
[1] Definition of cardiac toxicity was not uniform						

eTable 4: Estimated and Reported Events and Hazard Ratios, by Trial and Treatment Group

		1 year Trastuzumab (events)			< 1 year Trastuzumab (events)			HR (95% CI)/90 % CI		Survival Rates	
Endpoint	Study	Estimated	Reported	Difference	Estimated	Reported	Difference	Estimated	Reported	Estimated	Reported
DFS	Pivot X[16]	339	345	-6	354	359	-2	1.08(0.93-1.25)	1.08(0.93-1.25)	Yes (all time points)	3y, 5y & 7y
	Joensuu H[13]	102	105	-3	139	140	-1	1.41(1.14-1.75)	1.39 (1.12-1.72)	Yes (all time points)	5y
	Mavroudis D[14]	17	17	0	28	28	0	1.57(0.86-2.87)	1.58 (0.86-2.10)	Yes (all time points)	NIL
	Earl H[17]	237	247	-10	255	265	-10	1.08 (0.93-1.25)	1.07 (0.93-1.24)	Yes (all time points)	4y
	Conte PF[12]	90	89	1	98	100	-2	1.12(0.88-1.42)	1.15(0.91-1.46)	Yes (all time points)	Nil
	Combined	785	803	-18	874	892	-15	1.14 (1.03-1.25)	NA	Yes (all time points)	NA
OS	Pivot X[16]	169	170	-1	187	186	1	1.13(0.92-1.39)	1.13(0.92-1.39)	Yes (all time points)	Nil
	Joensuu H[13]	43	44	-1	62	58	4	1.49 (1.07-2.06)	1.36 (0.98-1.89)	Yes (all time points)	5y
	Mavroudis D[14]	9	10	-1	7	8	-1	1.49 (0.55-4.02)	1.45(0.57-3.67)	Yes (all time points)	NIL
	Earl H[17]	149	156	-7	171	179	-8	1.15 (0.95-1.38)	1.14(0.95-1.37)	Yes (all time points)	4y
	Conte PF[12]	38	37	1	36	38	-2	1.03(0.70-1.50)	1.06(0.73-1.55)	Yes (all time points)	Nil
	Combined	408	417	-9	463	469	-6	1.17 (1.04-1.33)	NA	Yes (all time points)	NA

© 2020 Gulia S et al. *JAMA Network Open.*

eTable 5. Estimated Disease-Free Survival at Various Points Using Individual Patient Data From 5 RCTs

		Estimated disease-free survival			Reported disease-free survival	
**Time**	**Study**	**1-year trastuzumab (%, 95% CI)**	**<1-year trastuzumab (%, 95% CI)**	**Difference (%, 95% CI)**	**1 year**	**< 1 year**
1 year	Pivot X	97.03 (96.09-97.74)	95.77 (94.69-96.63)	1.26 (-0.03-2.55)		
	Joensuu H	99.17 (98.41-99.57)	98.88 (98.04-99.36)	0.299 (-0.54-1.12)		
	Mavroudis D	100	98.73 (96.11-99.59)	1.27 (0.17-2.71)		
	Earl H	99.21 (98.72-99.52)	99.11 (98.60-99.44)	0.1 (-0.46-0.66)		
	Conte PF	98.39(97.03-99.13)	98.38 (97.00-99.12)	0.01 (-1.41-1.43)		
	Combined	98.50 (98.15-98.78)	97.98 (97.58-98.31)	0.52 (0.03-1.01)		
2 year	Pivot X	93.96 (92.7-95.0)	91.41 (89.95-92.66)	2.55(-1.36-2.34)	93.8	91.1
	Joensuu H	97.04 (95.84-97.90)	95.96 (94.60-96.99)	1.08 (-1.09-2.08)		
	Mavroudis D	98.24 (95.38-99.34)	97.42 (94.34-98.83)	0.82(-2.26-3.24)		
	Earl H	95.71 (94.73-96.52)	95.50 (94.5-96.33)	0.21 (-0.78-1.76)	96.1	95.7
	Conte PF	96.12 (94.26-97.38)	94.44(92.31-96.00)	1.68 (-1.94-2.92)		
	Combined	95.60 (95.03-96.10)	94.34 (93.7-94.91)	1.26 (0.42-2.10)		
3 year	Pivot X	90.71 (89.21-92.01)	87.89 (86.21-89.37)	0.82 (-1.67-2.65)		
	Joensuu H	94.90 (93.39-96.07)	92.68 (90.92-94.11)	2.22 (-1.76-2.75)		
	Mavroudis D	95.62 (91.70-97.71)	93.36 (89.02-96.03)	2.26(-4.38-5.36)	95.7	93.3
	Earl H	92.51 (91.25-93.59)	91.42 (90.08-92.58)	1.09 (-1.25-2.23)		
	Conte PF	92.06(89.62-93.94)	91.16(88.61-93.16)	0.9 (-2.75-3.73)		
	Combined	92.05 (91.77-93.17)	90.67 (89.86-91.41)	1.38 (0.24-2.52)		
4 year	Pivot X	87.58 (85.88-89.08)	85.49 (83.69-87.11)	2.09 (-2.09-3.07)	88.8	86.1
	Joensuu H	92.5 (90.67-93.98)	90.01(87.96-91.73)	2.49(-2.40-3.38)		
	Mavroudis D	93.63 (88.94-96.37)	88.33 (82.69-92.22)	5.3(-6.69-7.67)		
	Earl H	89.82(88.35-91.11)	89.41(87.94-90.72)	0.41 (-1.85-2.83)	89.8	89.4
	Conte PF	90.12(87.45-92.24)	88.52 (85.68-90.82)	1.6 (-3.39-4.37)		
	Combined	89.82 (88.97-90.60)	88.23(87.33-89.07)	1.59 (0.22-2.96)		
5 year	Pivot X	85.25 (83.42-86.89)	83.52 (81.62-85.24)	1.72 (-2.44-3.42)	86.2	84.2
	Joensuu H	90.82 (88.75-92.52)	88.20 (85.94-90.12)	2.62 (-3.09-4.07)	90.5	88
	Mavroudis D	89.75 (83.35-93.77)	85.68 (79.23-90.24)	4.07 (-9.49-10.47)		
	Earl H	86.27 (84.51-87.84)	85.28 (83.48-86.91)	0.99 (-2.55-3.53)		
	Conte PF	87.39 (84.41-89.84)	85.38(82.18-88.05)	2.01 (-4.46-5.44)	88	85
	Combined	87.12 (86.15-88.02)	85.42 (84.41-86.38)	1.7 (0.01-3.39)		
	**p for non-inferiority, 0.0042**					

© 2020 Gulia S et al. *JAMA Network Open.*

eTable 6. Estimated Overall Survival at Various Points Using Individual Patient Data From 5 RCTs

		Estimated overall survival			Reported overall survival	
**Time**	**Study**	**1 year of trastuzumab (%, 95% CI)**	**< 1 year of trastuzumab (%, 95% CI)**	**Absolute Difference (%, 95% CI)**	**1 year**	**< 1 year**
1 year	Pivot X	99.82 (99.45-99.94)	99.11(98.52-99.46)	0.71 (0.21-1.20)		
	Joensuu H	100	100	0 (0-0)		
	Mavroudis D	100	99.58 (97.08-99.94)	0.42 (-0.41- 1.24)		
	Earl H	99.21(98.72-99.52)	99.12 (98.60-99.44)	0.09 (- 0.47-0.65)		
	Conte PF	99.84 (98.86-99.87)	100	- 0.16(-0.31-0.33)		
	Combined	99.65 (99.45-99.77)	99.40 (99.16-99.57)	0.25 (-0.004-0.504)		
2 year	Pivot X	98.62 (97.63-99.08)	97.11 (96.18 -97.81)	1.51 (0.51-2.51)		
	Joensuu H	99.45 (98.77-99.75)	98.31 (97.34-98.93)	1.14 (0.24-2.04)		
	Mavroudis D	100	99.58 (97.08-99.94)	0.42 (-0.44-1.28)		
	Earl H	98.02 (97.31-98.54)	97.52(96.74-98.12)	0.5 (-0.43-1.43)	98.9	98.7
	Conte PF	99.51 (98.50-99.84)	99.01 (97.82-99.56)	0.5 (-0.46-1.46)		
	Combined	98.64 (98.31-99.92)	97.87 (97.46-98.22)	0.77 (0.28-1.26)		
3 year	Pivot X	96.91 (95.95-97.64)	95.14 (93.99-96.08)	1.77 (0.40-3.14)		
	Joensuu H	98.47 (97.52-99.06)	97.50 (96.35-98.29)	0.97 (-0.31-2.25)		
	Mavroudis D	98.29 (94.78-99.44)	98.58 (95.63-99.54)	-0.29 (-2.91-2.33)		
	Earl H	96.02(95.06-96.8)	94.42(93.31-95.35)	1.6 (0.18 -3.02)		
	Conte PF	98.53 (97.2-99.23)	97.86 (96.34-98.75)	0.67 (-0.84-2.18)		
	Combined	97.06 (96.58-97.48)	95.84(95.28-96.34)	1.22 (0.49-1.95)		
4 year	Pivot X	94.97 (93.79-95.93)	93.58 (92.27-94.66)	1.39 (-0.28-3.06)		
	Joensuu H	97.12 (95.85-98.01)	96.45 (95.08-97.44)	0.67 (-1.08-2.42)		
	Mavroudis D	97.66 (93.89-99.12)	98.58 (95.63-99.54)	-0.92 (-4.29-2.45)		
	Earl H	94.79 (93.68-95.71)	93.79 (92.62-94.78)	1.0 (-0.73-2.73)	94.8	93.8
	Conte PF	95.93 (93.98-97.25)	96.63 (94.82-97.81)	- 0.7 (-2.99- 1.59)		
	Combined	95.61 (95.02-96.13)	94.59 (93.95-95.17)	1.02 (0.11-1.93)		
5 year	Pivot X	93.57 (92.25 - 94.67)	92.03 (90.59 - 93.26)	1.54 (-0.44- 3.52)		
	Joensuu H	95.86 (94.31 - 97.00)	94.69 (92.99 - 95.99)	1.17 (-1.23-3.57)	95.9	94.7
	Mavroudis D	96.76 (92.25 - 98.66)	97.78 (94.00 - 99.19)	-1.02 (-5.76-3.72)		
	Earl H	90.96 (89.43-92.27)	90.06 (88.51-91.41)	0.9 (-1.59-3.39)		
	Conte PF	95.11 (92.98 - 96.60)	95.12 (92.94 - 96.64)	-0.01 (-2.92- 2.92)	95.2	95.0
	Combined	93.46 (92.73-94.13)	92.39 (91.61-93.10)	1.07 (-0.14-2.28)		

© 2020 Gulia S et al. *JAMA Network Open.*

eTable 7: Estimated Events for the Subgroups in Each Trial

			< 1 year		1 year	
			**Events**	**N**	**Events**	**N**
**Age**	< 50	Earl H	94	677	93	657
		Joensuu H	90	352	63	364
		Mavroudis D	18	83	10	100
		Pivot X	132	594	129	600
	> 50	Earl H	171	1366	154	1388
		Joensuu H	63	731	29	724
		Mavroudis D	12	157	15	141
		Pivot X	227	1096	216	1090
**Estrogen status**	ER+	Conte PF	69	427	56	426
		Earl H	144	1411	148	1412
		Joensuu H	96	711	57	723
		Mavroudis D	19	165	9	156
		Pivot X	190	995	181	975
	ER-	Conte PF	42	199	34	201
		Earl H	121	632	99	632
		Joensuu H	58	374	34	366
		Mavroudis D	11	75	5	84
		Pivot X	169	695	164	715
**Nodal status**	N0	Conte PF	39	332	39	340
		Earl H	77	1019	70	1003
		Joensuu H	60	647	34	649
		Mavroudis D	15	40	2	61
	N1-2	Conte PF	30	194	30	189
		Earl H	65	486	53	479
		Joensuu H	46	322	26	320
		Mavroudis D	11	107	2	97
	N3	Conte PF	44	100	19	98
		Earl H	51	211	74	244
		Joensuu H	48	116	29	120
		Mavroudis D	10	93	5	83
**Stage**	I	Conte PF	28	264	27	245
		Joensuu H	36	427	19	430
	II	Conte PF	52	268	34	281
		Joensuu H	58	529	28	528
	III	Conte PF	38	91	21	100
		Joensuu H	37	129	23	131
**Timing of trastuzumab administration**	Sequential	Earl H	142	1091	165	1094
		Pivot X	163	729	150	718
	Concomitant	Conte PF	105	626	95	627
		Earl H	123	952	82	951
		Joensuu H	140	1085	105	1089
		Mavroudis D	28	240	17	241
		Pivot X	196	961	195	972
		Schneider BP	25	115	29	112

Abbreviations : N, no of patients; ER+, estrogen receptor positive; ER-, estrogen receptor negative; N0,node negative; N1-2, node positive (1-3); N3, node positive (4 or more)

© 2020 Gulia S et al. *JAMA Network Open.*
